# Long non-coding RNAs discriminate the stages and gene regulatory states of human humoral immune response

**DOI:** 10.1038/s41467-019-08679-z

**Published:** 2019-02-18

**Authors:** Xabier Agirre, Cem Meydan, Yanwen Jiang, Leire Garate, Ashley S. Doane, Zhuoning Li, Akanksha Verma, Bruno Paiva, José I. Martín-Subero, Olivier Elemento, Christopher E. Mason, Felipe Prosper, Ari Melnick

**Affiliations:** 1 Department of Medicine, Division of Hematology/Oncology, Weill Cornell Medicine, New York, NY 10021 USA; 2 Division of Hemato-Oncology, Center for Applied Medical Research (CIMA), University of Navarra, IDISNA, Ciberonc, Pamplona, 31008 Spain; 3 Department of Physiology and Biophysics, Weill Cornell Medicine, New York, NY 10065 USA; 4 The Bin Talal Bin Abdulaziz Alsaud Institute for Computational Biomedicine, Weill Cornell Medicine, New York, NY 10021 USA; 5 Department of Hematology, Clínica Universidad de Navarra, IDISNA, Pamplona, 31008 Spain; 6 Institut d’Investigacions Biomédiques August Pi i Sunyer (IDIBAPS), University of Barcelona, Barcelona, 08036 Spain; 7 The Feil Family Brain and Mind Research Institute, Weill Cornell Medicine, New York, NY 10021 USA

## Abstract

lncRNAs make up a majority of the human transcriptome and have key regulatory functions. Here we perform unbiased de novo annotation of transcripts expressed during the human humoral immune response to find 30% of the human genome transcribed during this process, yet 58% of these transcripts manifest striking differential expression, indicating an lncRNA phylogenetic relationship among cell types that is more robust than that of coding genes. We provide an atlas of lncRNAs in naive and GC B-cells that indicates their partition into ten functionally categories based on chromatin features, DNase hypersensitivity and transcription factor localization, defining lncRNAs classes such as enhancer-RNAs (eRNA), bivalent-lncRNAs, and CTCF-associated, among others. Specifically, eRNAs are transcribed in 8.6% of regular enhancers and 36.5% of super enhancers, and are associated with coding genes that participate in critical immune regulatory pathways, while plasma cells have uniquely high levels of circular-RNAs accounted for by and reflecting the combinatorial clonal state of the Immunoglobulin loci.

## Abstract (web summary)

Long non-coding RNAs (lncRNA) constitute a large fraction of human transcriptome, and are reported, individually, for context-specific regulatory functions. Here the authors expand our understanding by providing a systemic, unbiased annotation of lncRNA to establish an atlas of lncRNA landscape during the induction of human humoral immune responses.

## Introduction

The human transcriptome is extraordinarily complex, consisting of tens of thousands of long non-coding RNAs (lncRNAs) that far exceed the number of messenger RNAs (mRNAs) coding for proteins. LncRNAs are a highly heterogeneous group of functional molecules that have in common being longer than 200 nucleotides in length with little or no coding potential. The overwhelming abundance of lncRNAs in the human transcriptome was previously considered to be a consequence of transcriptional noise. However, recent studies indicate that many lncRNAs exhibit significant tissue- and cell-type specificity^1,2^, suggesting that lncRNAs have distinct cellular functions. Mechanistic studies indicate that lncRNAs are key regulators of biological processes including cell differentiation, development, and the immune system^3–6^. With the advent of new RNA-sequencing (RNA-seq) strategies, the annotation of human lncRNAs has remarkably expanded in the past few years^7,8^. However, the complete landscape of lncRNAs in the humoral immune response and their functional genomic characterization and links to chromatin features remains largely unexplored.

Humoral immunity is a multilayered process that involves activation and maturation of B cells. Germinal centers (GCs) are the focal point of this process. GCs form upon activation by the T cell-dependent antigen response, when naive B (NB) cells migrate to the interior of lymphoid follicles. The GC reaction is highly dynamic and features repeated cycling of B cells from the B cell-rich dark zone to the more heterogeneous light zone. Dark zone GC B cells are called centroblasts (CBs), which undergo repeated rounds of rapid proliferation and somatic hypermutation^9,10^. These cells eventually migrate to the light zone and become centrocytes (CCs) that undergo clonal selection and terminal differentiation to memory B cells (MEM) or plasma cells (PCs). PCs exiting the lymph nodes then migrate to the bone marrow to become long-lived PCs, specialized in the production and secretion of immunoglobulins (Igs)^9,11^. Although there is extensive experimental data regarding the molecular and cellular signals that control the proliferation and differentiation of B cells^12,13^, information on global transcription during the humoral immune response is limited.

Recently, Petri et al.^14^ analyzed the expression of lncRNAs in 11 discrete human B cell subsets using exon array-based technology. In this study, they detected 1183 lncRNAs associated with seven coding genes sub-networks related to distinct stage of B cell development, including terminal differentiation. In a subsequent study, Brazão et al.^15^ reported a catalog of 4516 lncRNAs expressed across 11 mouse B cell populations, including stages of terminal B cell differentiation using the stranded polyA+ RNA-seq strategy. They identified 1878 novel intergenic lncRNAs, some of which were related to histone modification marks associated with enhancer or promoter regions. These studies point to importance of fully characterizing the full transcriptome of B cells as they undergo the GC reaction and subsequent terminal differentiation. When taken together with the rapidly shifting chromatin landscape of B cells undergoing Ig affinity maturation, the lncRNA transcriptome could provide a more complete understanding of basic molecular immune mechanisms and the B cell context-specific transcriptome. Therefore, herein we set out to perform a full de novo annotation of the B cell non-coding transcription and its functional relationship with the epigenome and coding transcriptome. Our studies provide evidence that lncRNAs are specifically expressed in each stage of the humoral immune response and are transcribed from specific enhancer regions related to key stage -specific phenotype-driving genes.

## Results

### The human humoral immune B cell non-coding transcriptome

**Fig. 1:**
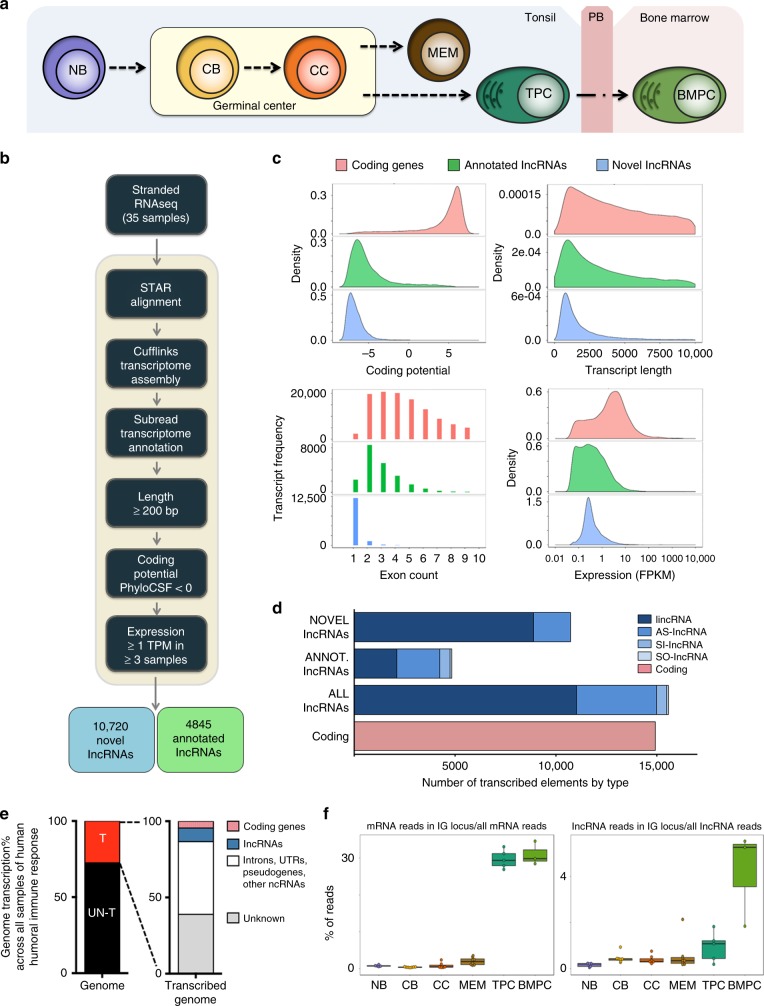
Identification of novel and previously annotated long non-coding RNAs (lncRNAs). **a** Schematic illustration of terminal differentiation of B cells from naive to plasma cells. **b** Workflow used to define and identify the novel and annotated lncRNAs expressed during the humoral immune response. **c** Coding potential, distribution of transcript lengths, distribution of number of exons per transcript, and expression levels for protein-coding genes (messenger RNA (mRNA)—red), previously annotated lncRNAs (annotated lncRNA—green), and novel lncRNAs (blue). **d** Number of lncRNAs and coding genes expressed. **e**, **f** Percentage of transcribed genome during the humoral immune response. **f** Box plots showing the percentage of coding genes or lncRNAs reads in Ig locus with respect to all coding genes or lncRNAs. Box plots show the median as center, first and third quartiles as the box hinges, and whiskers extend to the smallest and largest value no further than the 1.5× interquartile range (IQR) away from the hinges. NB: naive B cells; CB: centroblasts; CC: centrocytes; MEM: memory B cells; TPC: tonsillar plasma cells; BMPC: plasma cells from bone marrow of healthy donors

To characterize the lncRNA transcriptome of B cells reflecting the humoral immune response, we obtained tonsils and bone marrow of healthy human donors and used multiparameter fluorescence-activated cell sorting to isolate the distinct B cell populations. Based on the expression level of nine different surface antigens, we isolated NB cells, CBs, CCs, MEM cells, tonsillar plasma cells (TPCs—these are plasmablasts), and bone marrow plasma cells (BMPCs) (Supplementary Fig. 1 and Fig. 1a). The ribo-depleted RNA from these cell populations (as described in Supplementary Fig. 1) were submitted to paired-end strand-specific RNA-seq (ssRNA-seq) and transcripts.

As expected for RNA-seq capturing the global transcriptome, the majority of the obtained reads mapped in introns and intergenic regions (Supplementary Fig. 2). To validate the selected populations, we examined the transcript abundance of genes used for fluorescence-activated cell sorting (FACS) and observed the expected patterns of differential expression. For example, the average expression of *CD10* was 9.7-folds higher in GCs compared to NB subpopulations, whereas *CD44* was expressed at lower level in GC-derived cells vs. NB cells or BMPCs (Supplementary Fig. 3). Moreover, GC master regulatory factors *BCL6* and *EZH2* were highly differentially expressed in GC B cells, whereas plasma cell master regulators *IRF4* and *PRDM1* were highly expressed in plasma cells (Supplementary Fig. 3).

To generate the full annotation of the B cell non-coding transcriptome beyond the currently annotated transcriptome, we performed de novo transcriptome discovery using the Cufflinks software^16^. As the criteria to distinguish novel expressed lncRNAs, we selected transcripts longer than 200 nucleotides with a TPM (transcripts per million) value of expression >1 in at least three of the analyzed samples and an overlap <75% of their length with repetitive elements. We used PhyloCSF to predict the coding potential of the newly detected transcripts, where a coding transcript would have a score >0 for the majority of its codons^17^ (Fig. 1b). Transcripts with coding potential >0 in any of its codons in any open reading frame (ORFs) were excluded. We also depleted the intronic and sense-overlapping transcripts (supplementary material). After filtering, our data yielded 10,720 novel lncRNAs and 4845 annotated lncRNAs, thus defining the extent of non-coding RNA in the B cell humoral immune response (Fig. 1b, Supplementary Data 1, and Supplementary Data 2).

As in the case of previously annotated lncRNAs, the B cell novel lncRNAs showed very little coding potential, lower overall transcript length, fewer exons, and lower average transcript abundance than protein-coding genes (Fig. 1c). Sixteen percent of these novel lncRNAs were classified as antisense lncRNAs and 84% as intergenic lncRNAs (lincRNAs) (Fig. 1d). Although each B cell subpopulation showed variability in the number of expressed lncRNAs, notably, the sum of novel and annotated lncRNAs expressed during the humoral immune response (15,565) was higher than the expressed coding genes (14,904; Fig. 1d and Supplementary Fig. 4). Taking into account the expressed lncRNAs and coding genes and also pseudogenes, other ncRNAs, and unknown transcripts that we detected, we found that 30% of the total human genome is transcribed when taking together the different stages of B cell differentiation (Fig. 1e). Yet, the percent of genome transcribed was not uniform and was instead significantly different across B cell populations (*p* = 0.001 in analysis of variance (ANOVA) comparing genome transcription fraction by cell type). Thus, 7–8% of the genome is transcribed in NB cells, CBs, CCs, and MEM cells and 4–5% in plasma cells (TPCs vs. NB cells, *p* = 0.001; BMPCs vs. NB cells, *p* = 0.02). Although plasma cells had higher ratio of transcription from the Ig loci (Fig. 1f), this does not affect the percent of genome transcribed because we did not detect a reduction in the number of well-defined lncRNAs. Overall, the B cell non-coding transcriptome is more extensive than the coding transcriptome and involves a significant fraction of the human genome, which suggests greater functional and molecular complexity among B cells than previously suspected.

### The GC reaction features major shifts in lncRNA expression

**Fig. 2:**
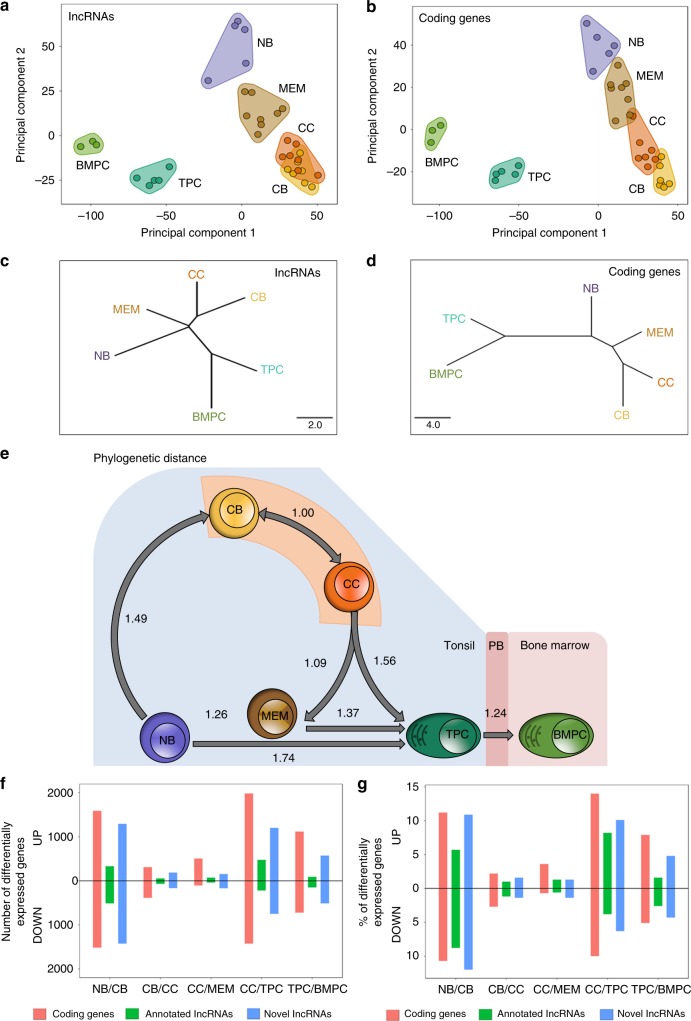
Differentially expressed long non-coding RNAs (lncRNAs) in each B cell subset. Unsupervised principal components analysis (PCA) of RNA-sequencing (RNA-seq) data for all **a** lncRNAs and **b** coding genes. Phylogenetic tree analysis of RNA-seq data for all **c** lncRNAs and **d** coding genes. **e** New scheme of B cell differentiation during the humoral immune response in which the memory B cells are positioned between the NB and GC B cells. The numbers show the distance between two of the B cell subpopulations. **f** The number of differentially expressed lncRNAs and **g** the percentage of differentially expressed novel lncRNAs, annotated lncRNAs, and protein-coding genes (with respect to total of expressed novel lncRNAs, annotated lncRNAs, and protenin-coding genes during the humoral immune response) between NB and CB, CB and CC, CC and MEM, CC and TPC, and TPC and BMPC. NB: naive B cells; CB: centroblasts; CC: centrocytes; MEM: memory B cells; TPC: tonsillar plasma cells; BMPC: plasma cells from bone marrow of healthy donors

We performed unsupervised analysis to compare and contrast lncRNA expression profiles among subpopulations of B cells. A principal component analysis (PCA) revealed robust segregation of B cell subsets (Fig. 2a). The first principal component distinguished NB cells, CBs, CCs, and MEM cells from plasma cells, whereas the second principal component distinguished NB and MEM cells from CBs and CCs. MEM cells occupied a space between GC and NB cells. A similar distribution was observed when considering either the known or novel annotated sets of lncRNAs (Supplementary Fig. 5a, b), or when performing PCA using coding genes (Fig. 2b). A phylogenetic analysis of lncRNAs, or coding genes, yielded similar polarity with MEM cells branching off from an intermediate point between NB cells and GC B cells, even though MEM cells arise in a step-wise manner after the GC reaction (Fig. 2c, d). TPCs were closer to B cells than BMPCs by both PCA and phylogenetic analysis. This phylogenetic relationship was also observed when considering protein-coding RNAs. These findings are consistent with the notion that the GC B cell non-coding and coding transcriptome represent a departure from the B cell differentiation program, which is restored upon exit from the GC reaction prior to terminal differentiation (Fig. 2e).

To measure the magnitude of lncRNAs transcriptome changes during the humoral immune response, we performed differential gene expression analyses between the sequential stages of the humoral response (see Methods section). Each of these transitions featured differential expression of non-coding RNAs. We observed 3719 lncRNAs (novel + annotated) differentially expressed between NB cells and CBs, 508 between CBs and CCs, 458 between CCs and MEM, 2776 between CCs and TPCs, and 1397 between TPCs and BMPCs (Fig. 2f, Supplementary Fig. 6a, b, and Supplementary Data 3). In all the comparisons, more than 55% of the differentially expressed lncRNAs were transcribed from intergenic regions (Supplementary Fig. 6c). Thus, during the human humoral immune response, the major shifts in lncRNA expression occur when NB cells transition to CBs and when CCs differentiate into plasma cells (Fig. 2g). A similar biphasic pattern was observed for both annotated and novel lncRNA as well as coding genes (Fig. 2f–g and Supplementary Fig. 6a, b).

### Striking immune-stage-specific expression of lncRNAs

**Fig. 3:**
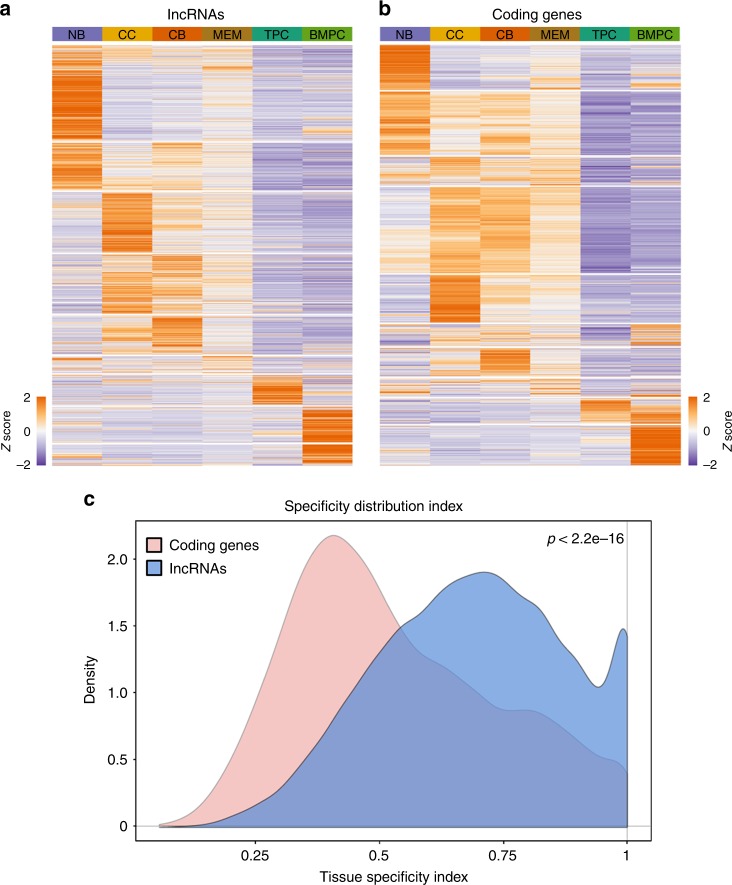
Specific expressions of long non-coding RNAs (lncRNAs) during human humoral immune response. **a** Heatmap showing all expressed lncRNAs in B cell subpopulations. **b** Heatmap showing all expressed protein-coding genes in B cell subpopulations. **c** Tissue specificity distribution index of lncRNAs and protein-coding genes in B cell subtypes (*p* < 2.2 × 10^−16^ by Wilcoxon's rank-sum test)

Visualization of expression patterns indicated that each B cell population features a unique lncRNA signature that distinguishes it from the preceding cell type (Fig. 3a, b) where BMPCs seem to have the most cell-type-specific and uniquely expressed lncRNAs, followed by NB cells (Supplementary Fig. 7a). Consistent with previously noted results, the largest shift in differentially expressed genes occurred between NB cells and CBs. In contrast, very few genes were differentially expressed between CCs and MEM cells. Overall, our studies revealed that a striking 57.8% of expressed lncRNAs manifested significant change in transcript abundance during the humoral immune response. Moreover, when comparing and contrasting lncRNA vs. coding genes, we observed that the specificity of lncRNAs signatures for each B cell subset as measured by the tissue specificity index tau^18^ was significantly higher than the specificity of coding genes (*p* < 2.2 × 10^−16^, Wilcoxon's rank-sum test) (Fig. 3c). Between lncRNAs, the intergenic lncRNAs showed increased tau scores compared to antisense lncRNAs (Supplementary Fig. 7b). Thus, lncRNAs are expressed in a more precisely stage-specific manner than coding RNAs.

### Dynamic regulation of lncRNA gene modules

**Fig. 4:**
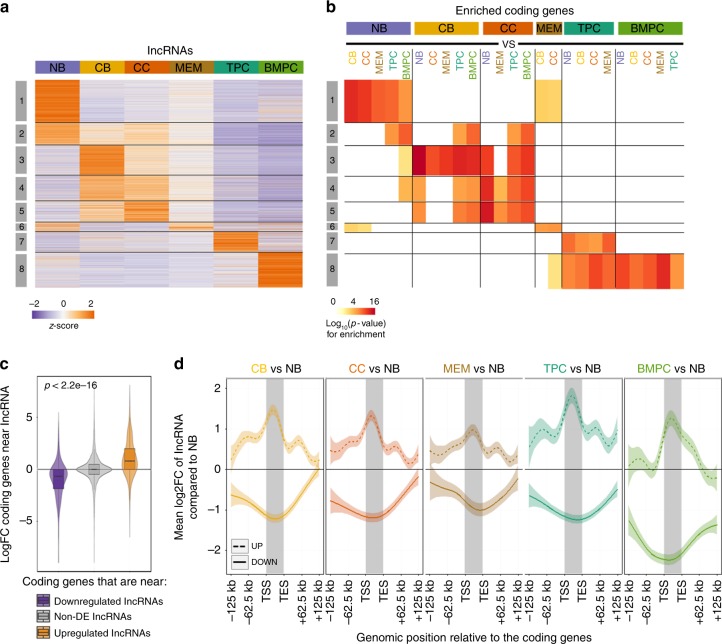
Dynamic expression of long non-coding RNAs (lncRNAs) in the germinal center (GC) reaction. **a** Heatmap showing *k*-means clustering of specific lncRNAs of each B cell subpopulation indicating dynamic modulation patterns of lncRNAs across different B cell subtypes. **b** Plot showing the expression of protein-coding genes that are adjacent to lncRNAs specifically expressed in each B cell subpopulations. The enrichment of differentially upregulated genes near lncRNA clusters is shown. **c** Plot showing the average expression of protein-coding genes that are adjacent to lncRNAs that are differentially expressed in comparison between two B cell subpopulations (*p* < 2.2 × 10^−16^ by Wilcoxon's rank-sum test). Box plots show the median as center, first, and third quartiles as the box hinges, and whiskers extend to the smallest and largest value no further than the 1.5× interquartile range (IQR) away from the hinges. **d** Expression and localization of lncRNAs in relation to protein-coding genes up- or down-regulated. The changes in lncRNAs expression are shown by their proximity to coding genes that are differentially expressed in different stages. NB: naive B cells; CB: centroblasts; CC: centrocytes; MEM: memory B cells; TPC: tonsillar plasma cells; BMPC: plasma cells from bone marrow of healthy donors

We next wished to identify groups of transcripts that follow defined trajectories during the humoral immune response. lncRNAs segregated into eight groups by *k*-means clustering on the normalized expression of the transcript across the six different cell types (see Methods section and Supplementary Data 1). These clusters of lncRNAs corresponded to transcripts (1) expressed in NB cells (1983 lncRNAs), (2) expressed in NB cells and their expression decreased progressively during subsequent stages (1004 lncRNAs), (3) uniquely expressed in CBs (1374 lncRNAs), (4) expressed in both CBs and CCs (1161 lncRNAs), (5) expressed in CCs at a higher level and decreased progressively during their differentiation (955 lncRNAs), (6) expressed in NB and MEM (403 lncRNAs), (7) uniquely expressed in TPCs (933 lncRNAs), and finally (8) uniquely expressed in long-lived BMPCs (1632 lncRNAs) (Fig. 4a). The expression patterns of these lncRNAs were validated by quantitative PCR (Q-PCR) in three newly isolated samples of each type of B cell subpopulations included in this study (Supplementary Fig. 8).

We then matched the lncRNAs from each cluster to nearby coding genes (see Methods section). Performing enrichment analysis for each cluster revealed that cell-stage-specific lncRNAs tend to be near coding genes that are also significantly differentially expressed and functionally relevant to each particular cell type (Fig. 4b and Supplementary Fig. 9). For example, lncRNAs expressed in NB cells (cluster 1) were close to genes upregulated in NB cells and implicated in lymphocyte activation and BCR signaling pathway. GC B cell-specific lncRNAs were related to antigen-dependent B cell activation genes and genes associated with lymphomas. Finally, the lncRNAs expressed specifically in TPCs were related to genes upregulated in plasma cells, targets of IRF4, and associated with multiple myeloma (MM) (Supplementary Data 4). These data support potential roles for lncRNAs in cell context-specific transcriptional patterning during the humoral immune response.

Globally, coding genes in close vicinity to lncRNAs that were either upregulated or downregulated during the humoral immune response showed significantly higher or lower expression, respectively (*p* < 2.2 × 10^−16^, Wilcoxon's rank-sum test), to coding genes near non-differentially expressed lncRNAs or coding genes that do not have any expressed lncRNAs in their upstream/regulatory regions (Fig. 4c). However this result does not necessarily imply that the majority of coding genes with cell-type-specific expression patterns have lncRNAs near them, as the data shown in Fig. 4c could be driven by a large number of lncRNAs clustered around a small number of key coding genes. We analyzed the transcriptional changes of all lncRNAs near coding genes differentially expressed in the comparison of two B cell subpopulations. We found that the lncRNAs tend to follow the same direction of change as nearby coding genes and this link is strongest around transcription start sites (TSSs) (Fig. 4d). These results suggest that the proximity of lncRNA could play a role in the regulation of the expression of coding genes, although as it has been described in recent studies that coding genes could also be regulated by functional elements of the genome and not only by the expression of non-coding elements^19^.

### Classification of B cell lncRNAs into functional subsets

**Fig. 5:**
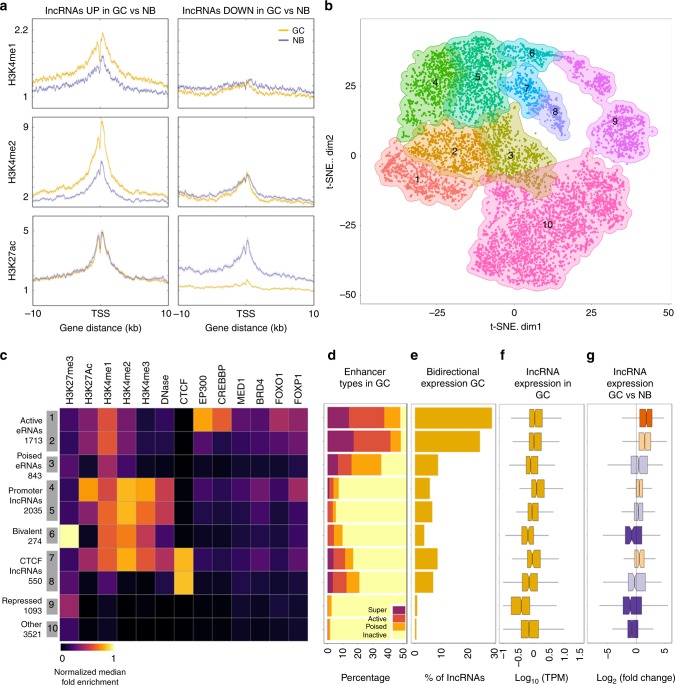
Categorization of long non-coding RNAs (lncRNAs). **a** Density plot of chromatin immunoprecipitation-sequencing (ChIP-seq) levels of H3K4me1, H3K4me2, and H3K27ac in lncRNAs differentially expressed between naive B (NB) cells and germinal center (GC) B cells. **b** *t*-distributed stochastic neighbor embedding (*t*-SNE) plot showing 10 different clusters of lncRNAs expressed in GCs and their association with ChIP-seq levels of H3K4me1, H3K4me2, H3K4me3, H3K27ac, H3K27me3, DNase, CTCF, EP300, CREBBP, MED1, BRD4, FOXO1, and FOXP1. **c** Heatmap of median of ChIP-seq enrichments in each of the 10 clusters of lncRNAs expressed in GC cells. **d** Percentage of enhancer types, **e** percentage of bidirectional expression, and **f** average expression of lncRNAs in each of the 10 clusters of lncRNAs expressed in GC cells and **g** with respect to NB cells. Box plots show the median as center, first and third quartiles as the box hinges, and whiskers extend to the smallest and largest value no further than the 1.5× interquartile range (IQR) away from the hinges

LncRNA expression and function is linked to chromatin features^6,20^. To gain better understanding of lncRNA regulation and functionality, we examined the chromatin state of their TSSs focusing specifically on the NB cell to GC transition, which features the most extensive lncRNA differential expression. For this analysis, we examined our previously published chromatin immunoprecipitation-sequencing (ChIP-seq) profiles for H3K4me1, H3K4me2, H3K4me3, and H3K27Ac in primary human NB and GC cells. Of note, whereas lncRNAs that were upregulated in GC vs. NB cells featured mainly gain of H3K4me1 and H3K4me2 in GCs. In contrast, lncRNAs regulated in GCs were generally associated with more impressive loss of H3K27Ac and to some extent H3K4me3 (Fig. 5a and Supplementary Fig. 10a). These findings provide a basis for future studies to mechanistically dissect mechanisms that control lncRNAs during the GC reaction.

To more thoroughly characterize the regulatory state of GC B cell lncRNA transcriptome, we next determined its association with a more extensive series of regulatory features and transcription factors based on datasets obtained in human GC or GC-derived B cells. These included ChIP-seq for H3K4me1, H3K4me2, H3K4me3, H3K27ac, H3K27me3, and DNase I-hypersensitive sites on NB and GC cells; chromatin factors CTCF, EP300, CREBBP, MED1, and BRD4; and transcription factors FOXO1 and FOXP1^13,21,22^ (GEO dataset GSE53601). The multi-dimensional data for lncRNAs were embedded in two dimensions by *t*-distributed stochastic neighbor embedding (*t*-SNE) and clustered using SamSPECTRAL for spectral clustering^23^. This analysis indicated that lncRNAs distribute into 10 distinct clusters in GC B cells based on their chromatin regulatory state (Fig. 5b, Supplementary Fig. 10b, and Supplementary Data 5). Clusters 1 and 2 enriched for H3K4me1, H3K4me2, H3K27Ac, and DNAse I, whereas cluster 3 enriched H3K4me1 (Fig. 5c and Supplementary Fig. 10c, d). Clusters 1 and 2 featured a higher percentage of enhancers (Fig. 5d), bidirectionally expressed lncRNAs (Fig. 5e), and higher expression in GC cells than in NB cells (Fig. 5f–g), the latter of which was also evident for cluster 3. Cluster 1 was also characterized by the enrichment of chromatin and TFs that are important for B cell differentiation. These characteristics suggest that these three clusters correspond to enhancer RNA (eRNAs). Clusters 4 and 5 enriched H3K4me1, H3K4me2, H3K4me3, and H3K27Ac, manifested a trend towards increased expression in GC B cells, and were mostly corresponding to lncRNAs expressed from promoter regions (Fig. 5c, d and Supplementary Fig. 10d). Cluster 6 enriched active marks H3K4me1, H3K4me2, and H3K4me3 in combination with H3K27me3 repressive mark and were generally repressed in GC B cells; thus, corresponding to likely bivalent chromatin lncRNAs (it is known that GC B cells acquire many new bivalent chromatin domains due to their upregulation of EZH2)^24^. Clusters 7 and 8 enriched for CTCF binding sites and perhaps are in some way associated with CTCF boundary or insulator functions. Cluster 9 was associated with H3K27me3 and represents silenced lncRNAs. Cluster 10 did not enrich for any of these features and are of unknown significance. Collectively this approach provides a functional atlas of the non-coding GC B cell transcriptome.

### Disproportionate eRNA abundance in B cell super-enhancers

Given the key role of enhancers in cell context-specific transcription, we next focused our attention on eRNAs and their potential association with the GC B cell regulatory state (Supplementary Data 5). Using our ChIP-seq data we identified 8274 and 9710 active (H3K4me1 and H3K27Ac) and 16,288 and 14,289 poised (H3K4me1) enhancer regions in primary human GC and NB cells, respectively. Among active enhancers, only 12.9% in GC cells and 8.6% in NB cells featured eRNA expression. We validated the specific expression of eRNAs by Q-PCR in NB cells, CBs, and CCs isolated from three new tonsillar samples (Supplementary Figs. 11 and 12).

**Fig. 6:**
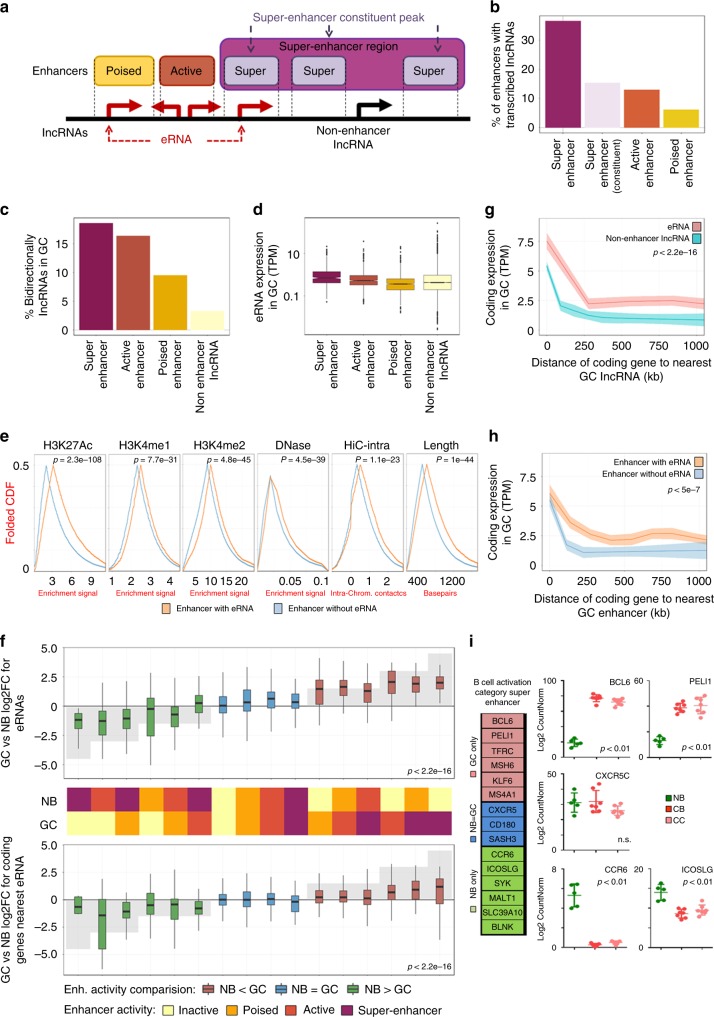
Enhancer RNAs in naive (NB) and germinal center (GC) B cells. **a** Cartoon showing the definition of enhancer, constituent enhancer, and super-enhancer regions. **b** Percentage of enhancer and super-enhancer regions with enhancer RNA (eRNA) transcription in GCs. **c** Percentage of long non-coding RNAs (lncRNAs) transcribed bidirectionally from super-enhancer regions, active (H3K4me1/H3K27ac), poised (H3K4me1), or inactive enhancer (regions that lack H3K4me1 in one cell type but not the other) regions in GCs. **d** Expression levels of lncRNAs transcribed from super-enhancer regions, active, poised, or inactive enhancer regions in GCs. **e** Mountain plots (folded cumulative distribution function (CDF)) of H3K27Ac, H3K4me1, H3K4me2, DNase hypersensitivy, normalized HiC contacts, and length of enhancer and super-enhancer regions with eRNA transcription compared to enhancer and super-enhancer regions without eRNA transcription. Mountain plots are created by folding the empirical cumulative distribution function around the median. Statistical significance was tested by Wilcoxon's rank-sum test. **f** Expression levels of eRNAs transcribed and coding genes closer from super-enhancer regions, active, poised, or inactive enhancer regions in GC or NB cells (*p* < 2.2 × 10^−16^ for both terms for NB and GC enhancer status, *p* < 0.0073 for interaction term between both terms using analysis of variance (ANOVA)). **g** Expression of coding genes with respect to distance to the nearest eRNAs or non-enhancer lncRNAs in GCs (*p* < 2.2 × 10^−16^ for interaction term of nearby lncRNA type with distance in ANOVA). **h** Expression of coding genes associated with the distance to enhancer regions with and without transcription of eRNAs in GCs as indicated (*p* < 5 × 10^−7^ for interaction of enhancer transcription term with distance in ANOVA). **i** Gene ontology enrichment of coding genes related to eRNAs in NB cells or GCs. NB: naive B cells; GC: germinal centers. ANOVA test was used for statistical analysis. Box plots show the median as center, first and third quartiles as the box hinges, and whiskers extend to the smallest and largest value no further than the 1.5× interquartile range (IQR) away from the hinges

We further identified 846 and 641 super-enhancer regions in GC and NB cells containing 4487 and 3757 constituent peaks, respectively (Fig. 6a). The abundance of eRNAs was increased to 15.3% in GC cells and 9% in NB cells in the case of super-enhancer constituent enhancers, and 36.5% in GC cells and 27.4% in NB cells in super-enhancer regions (Fig. 6b and Supplementary Fig. 13a). Super-enhancer regions also showed a higher percentage of bidirectional transcription of lncRNAs and higher expression of these lncRNAs (Fig. 6c, d and Supplementary Fig. 13b, c). Although super-enhancer regions are by definition longer than standard enhancers (Supplementary Fig. 13c), their length does not explain the higher percentage of transcribed lncRNAs. To test whether the increased ratio of transcription in super-enhancer regions is attributable to their longer length, we performed a random sampling test. Using the longer super-enhancer region enhancer lengths, numbers, and chromosomal distribution of our enhancer peaks, we generated random genomic coordinates and calculated the fraction of these peaks that were overlapping with an expressed lncRNA (same method used for enhancer transcription). This random sampling process was performed 1000 times, to test whether these artificially generated long regions would have higher ratio of transcription comparable to what is observed for real super-enhancer regions. None of the random samples matched the real ratio of transcribed enhancers (random peaks had median of 4.0% and maximum of 6.0% ratio of transcription in the 1000 permutations, whereas the real super-enhancer region eRNA transcription is 36.5%), suggesting that higher eRNA presence is not likely due to chance. The same pattern is also present in poised and active enhancers, even though they have approximately the same length, the rate of transcription is 2.1X in active enhancers compared to poised (Supplementary Fig. 13d).

Of particular note, we found that both enhancer and super-enhancer regions with eRNA transcription presented significantly higher levels of H3K27Ac, H3K4me1, H3K4me2, and DNase hypersensitivy, and a greater abundance of 3D contact points (based on primary human GC B cell HiC^21^) compared to enhancer and super-enhancer regions without eRNA transcription (Fig. 6e). Expression change of eRNAs between NB cells and GCs was significantly correlated with enhancer characteristics (*p* < 2.2 × 10^−16^ for both NB and GC enhancer status, *p* < 0.0073 for interaction term between both terms using ANOVA, Fig. 6f). eRNA transcript abundance at super-enhancers was associated with their higher levels of H3K27Ac and lower level of H3K27me3 as compared to standard enhancers (Supplementary Fig. 13e). The expression level of protein-coding genes close to enhancers was higher in GCs when the enhancer of super-enhancer regions had greater activity in GC cells than in NB cells (and vice versa) (Fig. 6f).

### Proximity to eRNAs are linked to higher transcription

**Fig. 7:**
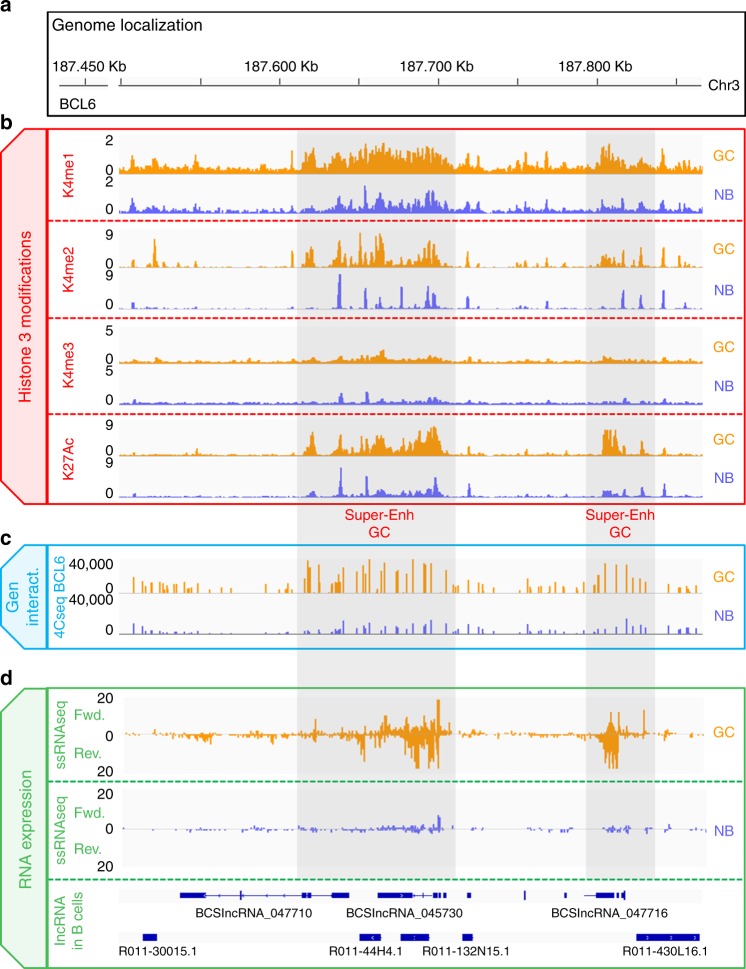
Long non-coding RNA (lncRNA) expression from *BCL6* enhancer region in the germinal center (GC) cells and naive B (NB) cells. **a** Genome localization of *BCL6* enhancer region. **b** Chromatin immunoprecipitation-sequencing (ChIP-seq) levels of H3K4me1, H3K4me2, H3K4me3, and H3K27ac in *BCL6* enhancer region in GC and NB cells. **c** 4c-BCL6-seq levels in *BCL6* enhancer region in GC and NB cells. **d** Stranded RNA-seq data in *BCL6* enhancer region in GC and NB cells

We next investigated whether the presence of eRNAs is linked to differential expression of protein-coding genes. Strikingly, we found that the protein-coding genes closer to eRNAs were the most highly expressed genes (Fig. 6g and Supplementary Fig. 13g). We did not detect a similar strong effect in the case of lncRNAs transcribed from non-enhancer regions (Fig. 6g and Supplementary Fig. 13g; *p* < 2.2 × 10^−16^ for interaction term of nearby lncRNA type with distance in ANOVA, or alternatively, *p* < 9 × 10^−11^ for log 2 fold-change (FC) difference between genes that are <250 kb away from eRNAs and non-enhancer lncRNAs). Similarly, the protein-coding genes closer to enhancer regions with eRNA transcription showed higher expression than those coding genes closer to enhancer regions without eRNAs (Fig. 6h and Supplementary Fig. 13h; *p* < 5 × 10^−7^ for interaction of enhancer transcription term with distance in ANOVA). In addition, super-enhancer regions with eRNA transcription were associated with critical genes linked to B cell receptor signaling, B cell activation, and humoral immune response (Supplementary Data 6 and 7). Examples in NB cells include *CCR6* and *ICOSLG*, and *PELI1* and *BCL6* (Figs. 6i and 7) in GC cells.

### circRNAs are derived from Ig genes in plasma cells

**Fig. 8:**
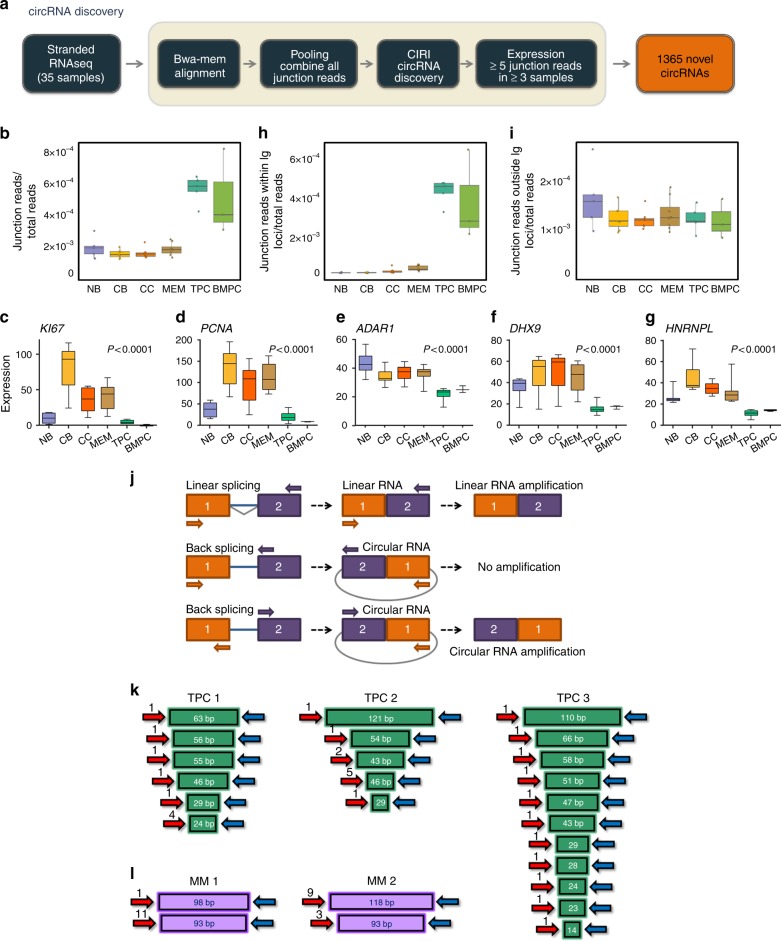
Identification of circular RNAs (circRNAs) during the human humoral immune response. **a** Scheme of pipeline used to define and identify the putative circRNAs expressed during the humoral immune response. **b** Box plots showing the average expression of total junction reads from circRNAs. **c** Box plots showing the expression level of *MKI67*, **d ***PCNA*, **e** *ADAR1*, **f** *DHX9*, and **g** *HNRNPL* genes in different B cell subpopulations. Analysis of variance (ANOVA) test was used for statistical analysis. **h**, **i** Box plots showing the average expression of junction reads derived from immunoglobulin (Ig) genes and junction reads derived from non-Ig genes. **j** Schematic representation of the circRNA formation by back splicing circularization and primers designed to properly amplify by PCR these circRNAs. **k**, **l** Cloning and sequencing results of a circRNA derived from Ig locus in normal tonsillar plasma cells or plasma cells from multiple myeloma patients as indicated. Box plots show the median as center, first and third quartiles as the box hinges, whiskers extend to the smallest and largest value no further than the 1.5× interquartile range (IQR) away from the hinges. NB: naive B cells; CB: centroblasts; CC: centrocytes; MEM: memory B cells; TPC: tonsillar plasma cells; BMPC: plasma cells from bone marrow of healthy donors; Ig: immunoglobulin genes; MM: multiple myeloma

Finally, we examined the complement of circular RNAs (circRNAs) in our B cell subsets using the CIRI algorithm^25^, and identified 1356 putative circRNAs expressed during the humoral immune response (Fig. 8a and Supplementary Data 8). The highest level of circRNAs was observed in plasma cells (Fig. 8b). Recent studies suggest that circRNAs are negatively correlated with proliferation^26,27^. Indeed, in the case of human plasma cells (TPCs and BMPCs), we observed this negative correlation between the expression of circRNAs and genes related with cell proliferation like *MKi67* and *PCNA*, but not in the case of NB cells (Fig. 8c, d). We further examined whether there was a correlation between the expression of circRNAs and RNA-binding proteins that have been linked recently to circRNA biogenesis, like *ADAR1*, *DHX9*, and *HNRNPL* that plays a negative regulation in the biogenesis of these non-coding elements^26–29^. We observed higher levels of circRNAs in those cells with lower levels of *ADAR1*, *DHX9*, and *HNRNPL* (Fig. 8e, g). These results suggest that, as has been described in other studies^26–29^, these RNA-binding proteins could participate in the regulation of the expression of the circRNAs during the human humoral immune response.

Notably, the circRNAs with by far the highest levels of expression in plasma cells were derived from Ig genes. Indeed the higher circRNA observed in plasma cells is fully accounted for by Ig transcripts (Fig. 8h and Supplementary Fig. 14). The circRNAs derived from non-Ig genes exhibited similar average expression levels in all B cell subpopulations of the humoral immune response (Fig. 8i). Although the circRNAs (Fig. 8h), protein-coding genes (Supplementary Fig. 15a, b), and lncRNAs (Supplementary Fig. 15c, d) derived from Ig locus were expressed to a greater extent in plasma cells, the expression of genes derived from Ig locus did not change the average expression pattern of protein-coding genes (Supplementary Fig. 15e) and lncRNAs (Supplementary Fig. 15f). We observed high concordance between the RNA-seq data and the results obtained by Q-PCR in three newly isolated samples of each type of B cell subpopulation included in this study (Supplementary Fig. 15g and h).

Next, we cloned the amplification products (Fig. 8j) of circRNA obtained from TPCs in a TOPO-TA vector to verify the sequence of the junction region of the circRNAs derived from Ig genes in plasma cells obtained from three different healthy donors. We observed different length sequences corresponding to the junction region of Ig locus circRNA (Fig. 8k). These distinct junction lengths likely reflect the polyclonal nature of normal plasma cells. In contrast, when we amplified the same circRNA junction region from plasma cells of patients with Multiple Myeloma (MM), we detected a single predominant sequence from each patient, consistent with the clonal nature of these neoplasms (Fig. 8l). These results indicate that each rearranged Ig gene results in a specific Ig-derived circRNA, and are indicative of plasma cell clonality.

## Discussion

In this study, we provide the first comprehensive atlas of lncRNA and circRNA expression during the human humoral immune response. We identified 11,495 previously non-annotated lncRNAs featuring massive changes of their expression patterns during the human humoral immune response. These lncRNAs significantly expand the number of formerly defined and expressed lncRNAs in human B cells. Contrary to the long-held view that <2% of the human genome is transcribed, the findings of our study indicate that the lncRNAs in each of the B cell subpopulations are transcribed from a higher proportion of the human genome compared to the protein-coding genes^1,2^. As in other human tissues^3,4^, the expression of lncRNAs was highly cell type specific even within subsets of fully mature B cells, and marking the identity of cell subsets that undergo functional transitions and terminal differentiation of B cell lineage during the humoral immune response with more precision than the coding genes. Furthermore, lncRNAs expressed specifically in each B cell subset were transcribed from genomic regions located close to protein-coding genes that are enriched and relevant for the function of each B cell subpopulations, suggesting critical *cis* functionality of lncRNAs in the regulation of protein-coding genes during the humoral immune response.

Our studies showed that the expression of lncRNAs in B cell subpopulations strongly correlated with chromatin features such as histone modifications, chromatin architecture, and transcription factors, similar to previously reported studies^6,15,20,30^. These characteristics allowed us to classify lncRNAs expressed during the human humoral immune response in six different novel functional groups: eRNAs, promoter lncRNAs, bivalent lncRNAs, repressive lncRNAs, CTCF lncRNAs, and other lncRNAs. Although eRNAs exhibited the expression in B cell subpopulations, especially those that were transcribed from super-enhancer regions, these eRNAs were detected from a relatively low percentage of active enhancer regions. Only 8.6% of the active enhancer regions led to eRNA expression in our study, increasing this percentage to 36.5% in the case of super-enhancer regions. It is possible that this low detection of eRNAs in enhancer regions could be a consequence of the RNA-seq strategy used in our study. Although this hypothesis is not completely ruled out, in our study we detected very consistent differences in the chromatin organization structure between the enhancer with transcription of clear eRNA and enhancer without transcription of eRNA, suggesting that eRNAs are transcribed from certain enhancer regions active in the genome of each cell. It is noteworthy that those enhancer or super-enhancer regions with an eRNA transcription featured higher enrichment for activating histone modifications and 3D contacts (more DNAse and HiC contacts) than enhancer regions without eRNA transcription. This suggests that these enhancer regions need more chromatin accessibility and interactivity and eRNAs transcription to fulfill their regulatory functions. Additionally, the enhancer or super-enhancer regions with eRNA transcription were associated with protein-coding genes that play an essential role in B cell differentiation, such as BCL6 in the case of GC cells. Based on these observations, our studies suggest that eRNAs are key components of the chromatin regulatory machinery essential for the transcriptional control of cell key context-specific protein-coding genes. Perhaps, these eRNAs are important for contributing to the bridging or looping between regulatory enhancer regions of the genome^31,32^ that are crucial to establish the GC phenotype during the humoral immune response.

Another interesting group of novel functional lncRNAs defined in our study were those enriched by CTCF in GC cells. Recently, it has been shown that CTCF orchestrates the GC transcriptome and that it plays a very important role in the initiation and maintenance of the GC reaction^33^. In this sense, our results suggest that, like the coding genes regulated by CTCF, those lncRNA enriched by CTCF in GC cells could play a relevant role in the regulation of essential genes or pathways implicated directly in the initiation and proliferation reaction of GC cells. In the opposite way, we also identified a novel functional group of bivalent lncRNAs, enriched by H3K4me3 and H3K27me3. EZH2, H3K27 methyltransferase, mediates the GC proliferation and somatic hypermutation through the formation of bivalent chromatin domains in critical coding gene promoters, leading to a transient silencing of B cell differentiation and cell cycle checkpoint genes^13^. All these results globally indicate that there are different functional groups of lncRNAs that would be involved in the initiation, activation, regulation of essential genes, and routes for the correct human humoral immune response.

We also identified 1356 novel putative circRNAs expressed during the humoral immune response, showing the highest mean expression values in human plasma cells (TPCs and BMPCs). As in the case of neuronal differentiation, the expression of circRNAs was found to increase during terminal B cell differentiation. However, we believe that it is unlikely that the high expression of the circRNAs can be attributed solely to a process of accumulation as consequence of a low rate of cell proliferation. As reported previously^26–29^, we found a negative correlation between the expression of the circRNAs and the expression of RNA-binding proteins *ADAR1*, *DHX9*, and *HNRNPL*. Since depletion of these genes was previously shown to increase the expression of specific circRNAs^26–29^, it raises the possibility that *ADAR1*, *DHX9*, and *HNRNPL* might also be an important regulator of circRNAs' biogenesis during terminal B cell differentiation. Interestingly, our studies demonstrated that the explosive and cell-specific expression of circRNAs in human plasma cells predominantly occurred from the Ig genes, which are the Achilles heel of the plasma cells that serve as cellular factories for Ig synthesis. In the case of plasma cells obtained from healthy donors, we detected distinct lengths of the junction region of Ig-circRNAs. However, we found a single predominant sequence from the junction region of circRNA derived from Ig locus in monoclonal gammopathy like MM. These results have allowed us to hypothesize that the tight expression of the circRNAs derived from the Ig locus could occur, as described in the case of circRNAs derived from chromosomal translocations^34^, as a consequence of the massive and exquisitely controlled rearrangement of the Ig that occurs during the humoral immune response culminating in the plasma cell. Therefore, the Ig locus leads to a high expression of both linear mRNAs as circular non-coding RNAs in human plasma cells. This raises the possibility that Ig-derived circRNAs may in some way play an important role in the identity and function of plasma cells. CircRNAs have been previously shown to be transcribed from key genes with high expression, participating in the regulation of the expression of its host gene^35,36^. In the case of the Ig locus, the expression of the circRNAs may represent a novel regulatory mechanism for the Ig locus during the humoral immune response.

Taken together, our studies mapping the full non-coding transcriptome during the humoral immune response show these to be tightly linked to the various transitions that B cells undergo upon immune activation and suggest that lncRNAs contribute to the regulation of essential genes during this process. Although the exact function and implication of each of the lncRNAs detected in B cell subpopulations is unknown, our studies provide the basis for future studies to explore the function and contribution of lncRNAs during the human humoral immune response, especially in two of the essential steps of terminal B cell differentiation: the B cell activation when the NB cells enter the GC and the final establishment of long-lived plasma cells. In this way, our atlas of long non-coding RNAs could provide valuable insights on the role that lncRNAs play in each of the steps of B cell differentiation and whether their alteration have implications as a non-coding oncogenes or tumor suppressors in human tumors derived from these cells, such as diffuse large B cell lymphomas or MM.

## Methods

### FACS isolation of B cell subpopulations

NB cells, CBs, CCs, MEM cells, and plasma cells (TPCs) were isolated from human tonsils of healthy donors and (BMPCs) from bone marrow of healthy donors by multiparameter FACS using the expression level of nine different surface antigens as previously described^37^. Healthy donor samples were provided by the Biobank of the University of Navarra and were processed following standard operating procedures approved by the local Ethics and Scientific Committee. The following monoclonal antibody combination was used for the cell isolation from tonsils: CD45-OC515 (Clone HI30, Immunostep, Salamanca, Spain); CD20-Pacific Blue (Clone 2H7, BioLegend, San Diego, CA, USA); CD44-APCH7 (Clone G44-26, Beckton Dickinson, Durham, NC, USA). CD10 PE-Cy7 (Clone HI10a Beckton Dickinson, Durham, NC, USA); CD38-FITC (Clone LD38, Cytognos, Salamanca, Spain); CXCR4-PE (Clone 12G5, Beckton Dickinson, Durham, NC, USA); CD27-APC (Clone L128, Beckton Dickinson, Durham, NC, USA) and CD3-PerCP-Cy5.5 (Clone SK7, Beckton Dickinson, Durham, NC, USA). BMPCs were FACS (FACSAria II, Becton Dickinson Biosciences, Durham, NC, USA) from human bone marrow of healthy donors using CD38-FITC (Clone LD38, Cytognos, Salamanca, Spain); CD138-BV421 (Clone MI15, Beckton Dickinson, Durham, NC, USA) and CD27-BV510 (Clone 0323, BioLegend, San Diego, CA, USA). Monitoring of instrument performance was performed daily using the Cytometer SetupTracking (CST; BBeckton Dickinson, Durham, NC, USA) after laser stabilization.

### Strand-specific RNA-seq library preparation and sequencing

Total RNA from all B cell subpopulations was isolated using Trizol extraction method (Life Technologies), purified by RNeasy MinElute spin column (Qiagen) and treated with DNase I (Thermo Fisher) following the manufacturer’s instructions. Total RNA was quantified using NanoDrop Specthophotometer (NanoDrop Technologies). High-quality purified total RNA (300–600 ng) from each sample was used for library preparation according to the user's manual of Truseq Stranded Total ribo-depleted RNA sample preparation kit (Illumina). Library quality was assessed using Agilent 2100 Bioanalyzer (Agilent Technologies) and the quantity was determined using Qubit (Life Technologies). Strand-specific RNA libraries were multiplexed (four samples per lane) and the sequencing was done with Illumina HiSeq 2500 (Illumina) as 50 base paired-end runs.

### Quantitative RT-PCR

RNA was prepared as previously described. Complementary DNA (cDNA) was synthesized using Verso cDNA kit (Thermo Fisher) following the manufacturer’s instructions. Detection was done using Fast SyberGreen on 7900HT Fast Real-Time PCR System with 384-Well Block Module thermal cycler from Applied Biosystems and using the following conditions: initial step of 20 s at 95 °C followed by 40 cycles of 1 s at 95 °C and 20 s at 60 °C. The relative expression of each gene was quantified by the Log 2^(−ΔΔCt)^ method using the gene *GAPDH* as an endogenous control. All results are shown as the average of three independent experiments. Quantitative RT-PCR (Q-RT-PCR) primers for specific novel and annotated lncRNAs, eRNAs, and circRNAs are listed in Supplementary Table 1.

### circRNA sequencing

Amplification product obtained by Q-RT-PCR for circRNA-3 in three new samples of TPCs and plasma cells from two patients with MM were subcloned into pCR^®^ 4-TOPO^®^ plasmid using TOPO-TA Cloning^®^ Kit for Sequencing (Life Technologies) and transformed into *Escherichia coli* according to the manufacturer’s recommendations. Colonies with recombinant plasmids containing the described PCR products were screened by digestion with *Eco*RI (Amersham Biosciences). Candidate plasmid clones were sequenced using T7 and T3 universal forward and reverse primers.

### lncRNA and circRNA discovery

A two-step alignment procedure was performed with STAR v2.4^38^ to remove any potential ribosomal RNA leftover from the ribodepletion step. A first pass alignment was done against human ribosomal sequences allowing multi-mapping. Unmapped reads from the first step were then aligned to hg19 human reference using GENCODE v19^39^ junction points. Cufflinks v2.2.1^16,40^ was run on the resulting alignment files to create de novo transcriptome assembly specific to each sample using strand-specific settings and GENCODE v19 as a database. Cufflinks outputs for all of the samples were then merged with themselves and GENCODE database using cuffmerge. The resulting assembly was then filtered to only novel intergenic and antisense transcripts, removing isoforms for known genes as well as novel intronic sense-overlapping transcripts due to challenges in separating them from transcription artifacts. R^41^ and GNU parallel^42^ was used to facilitate the analysis.

The novel transcripts were then filtered for minimum length of 200. Coding potential of each transcript was checked using phyloCSF^17^ on all three ORFs of the transcript strand. Maximum score along the length of the transcript across all three ORFs was used as the maximum coding potential, and novel transcripts that had a coding potential score >0 were filtered out.

Subread featureCounts^43^ was used to annotate each sample to the merged transcript annotation. Resulting counts were normalized to library size using number of mapped reads to each sample by DESeq2^44^, and converted into Transcripts-pre-million (TPM)^45^. Any transcript (including coding genes, lncRNAs, and novel transcripts) that was expressed in at least three samples with ⩾1 TPM was included for the study.

We have used Human BodyMap v2, Human lincRNA, and GENCODE v28 references to annotate the transcribed elements for Fig. 1e. Coding exons of genes annotated as “Protein coding”, “Immunoglobulin variable chain,” and “T cell receptor” genes from Gencode and The International Immunogenetics Information System (IMGT) are classified as coding genes. Genes that are annotated as lincRNAs, lncRNAs overlapping introns or exons in the sense strand and on the antisense strand are classified as lncRNAs. Remaining regions are classified as other ncRNAs, which include introns and untranslated regions; Rfam- and miRbase-derived miRNAs, rRNAs, scRNAs, snRNAs, snoRNAs, sRNAs, scaRNAs, ribozymes and mitochondrial rRNA and tRNAs, pseudogenes, transposable elements and retrotransposed sequences, processed transcripts without an ORF, and other annotated regions. Any transcription that does not fall under any of the previously known annotations are defined as “Other” and may include lowly expressed uncharacterized ncRNAs as well as transcriptional and potential alignment artifacts.

Unsupervised clustering of the samples was done by PCA and phylogenetic analysis. PCA included in the manuscript were performed using the TPM values of the top 10% most variable genes (standard deviation across the samples). PCA on all genes without filtering yielded similar results (data not shown). Phylogenetic analysis was performed on a distance matrix created by log transforming the TPM values and calculating pairwise Euclidean and Pearson’s distance between each sample. The character matrix was generated using neighbor joining algorithm on the distance matrix and bootstrapping the result 100 times on resampled values using the ape package in R^46,47^. Both distances resulted in similar trees, and Supplementary Figure 4 shows the Euclidean distance. The analysis was repeated on a group level by comparing all pairwise distances between the members of two groups (e.g., between all NB samples and all CB samples) and taking the median and recalculating the bootstrapped neighbor joining tree. Results are shown in Fig. 2.

Differential expression was calculated with DESeq2 by pairwise comparisons of all cell types. Differentially expressed genes were selected by taking the genes with an absolute log 2(Fold Change) (log 2FC) of 1.5 and *q* value of <0.01.

circRNA discovery was performed by pooling all the samples and using BWA-MEM^48^ to align to hg19 reference. CIRI^25^ was run on the pooled sample to discover circRNAs. circRNA expression was quantified by matching the junction reads in the pooled data to their original sample. circRNAs that had ⩾5 junction points in at least three samples were included for study.

### Biological pathway enrichment analysis

Each lncRNA was associated to nearby coding genes by intersecting the lncRNA coordinates by the regulatory region of the coding genes. Regulatory region coordinates were defined by basal plus extension association in GREAT^49^ for each gene. This many-to-many lncRNA-coding gene association was used for all of the following analyses.

Gene expression was averaged by taking the median of TPM values for each cell type. lncRNAs were then clustered by *k*-means clustering^50^ with *k* = 8 using the group medians (Fig. 4A). Enrichment of pathways for each cluster were calculated by taking the nearby coding genes for every lncRNA belonging in a cluster and performing enrichment analysis by ACSNMineR^51^. All of the differentially expressed genes, custom curated list from the literature and MSigDB^52^ genesets were used as database. *P*-values as calculated by ACSNMineR were corrected with Benjamini–Hochberg procedure^53^. Pathways were filtered for *q* < 0.001 and ranked by −log10(*q* value) for each cluster. Top cell-type-specific signatures as calculated by tissue specificity index^18^ are shown in Fig. 4b and Supplementary Fig. 8.

Flanking region of coding genes (+−250 kb) that are differentially expressed between cell type transitions was intersected with the lncRNAs genomic coordinates. Mean log 2FC of lncRNA expression changes in the same comparison were plotted as a function of distance for both upregulated and downregulated coding genes (Fig. 4D).

### Chromatin marks and transcription factor binding sites

ChIP-seq data for histone modifications and transcription factors mentioned in the Results section were aligned to hg19 genome with BWA-MEM. Peaks and signal tracks were calculated using MACS v2^54^ by the FC enrichment of the binding compared to the input. For each lncRNA, a summary value of each signal track was calculated by taking the 90th percentile value in a window of +−1000 bp from the TSS. This was done to remove potential outliers and normalize for length.

Dimensionality reduction on the ChIP-seq data matrix was performed using *t*-SNE^23^. lncRNAs were separated into 10 clusters using spectral clustering with SamSPECTRAL^55^. For heatmap visualizations, the signal values for each feature were colored by mapping the feature value to a 0–1 range after removing the top and bottom 1%.

### Enhancers, super-enhancers, and eRNAs

Enhancers were called for NB cells and GCs separately using the H3K4me1 marks that were at least 2500 bp away from a coding gene TSS. H3K4me1 peaks that also contained an H3K27ac peak were annotated as “active enhancers”, whereas H3K4me1 peaks that did not overlap with H3K27ac peak were called as “poised enhancers”. Super-enhancer regions were called using the ROSE algorithm^56,57^ on H3K27ac data. Highly active enhancer regions that were merged together in a bigger region with ROSE are annotated as “super-enhancer regions”, whereas the specific enhancer peaks that lie within the general region are called “super-enhancer constituent peaks”.

eRNAs were called by taking the TSS+−1000 bp of single-exon intergenic lncRNAs and intersecting them with the enhancer peaks. Any eRNA within 2500 bp of a coding gene TSS was excluded. Activity of an eRNA was matched to the activity level of the enhancer (inactive region or poised enhancer, active enhancer, super-enhancer) for NB cells and GCs separately.

### Bidirectionality score

For a genomic coordinate *G*, window size of *w* and window count of *l*, two regions that flank *G* to the left (5′ of + strand) or right (3′ of + strand) excluding the center window were created as follows:

$$G_{{\mathrm{left}}} = \left[ {G-w \times l,\,G-w} \right],$$

$$G_{{\mathrm{right}}} = [G + w,\,G + w \times l].$$

The number of aligned reads that fall into either *G*_left_ or *G*_right_ from forward and reverse strands were summed up to give *F*_left_, *F*_right_ for forward strand, and *R*_left_, *R*_right_ for reverse strand. A dummy count *ε* was added to remove the exaggerating effects of very small counts on ratios. Bidirectionality score *B* was calculated as

$$B = {\mathrm{log}}_{10}\left( {\frac{{R_{{\mathrm{right}}} + \varepsilon }}{{R_{{\mathrm{left}}} + \varepsilon }} \times \frac{{F_{{\mathrm{left}}} + \varepsilon }}{{F_{{\mathrm{right}}} + \varepsilon }}} \right) - \left| {{\mathrm{log}}_{10}\left( {\frac{{R_{{\mathrm{right}}} + \varepsilon }}{{F_{{\mathrm{left}}} + \varepsilon }}} \right)} \right|,$$

where the ratio of forward and reverse expression would have a local maxima around the TSS of a bidirectionally expressed transcript. A regularization term was added so that transcripts with uneven expression on forward and reverse strands could be penalized.

For each intergenic lncRNA the bidirectionality score *B* was calculated with *w* = 100 and *l* = 7; lncRNAs with *B* > 0.5 were annotated as bidirectional.

### Reporting Summary

Further information on experimental design is available in the Nature Research Reporting Summary linked to this Article.

## Supplementary Information

Supplementary Information

Peer Review File

Description of Additional Supplementary Files

Supplementary Data 1

Supplementary Data 2

Supplementary Data 3

Supplementary Data 4

Supplementary Data 5

Supplementary Data 6

Supplementary Data 7

Supplementary Data 8

Reporting Summary

## Data Availability

Sequencing data that support the findings of this study have been deposited in GEO with the accession codes GSE114816 and GSE114803. Other sequencing data analyzed during this study are available in GEO with the accession codes GSE45982, GSE84022, GSE68349, GSE53601, and in ENCODE with accession code ENCSR000EIZ.
