# Supplementary material for: Long non-coding RNAs discriminate the stages and gene regulatory states of human humoral immune response
Source: Nat Commun. 2019 Feb 18;10:821. doi: 10.1038/s41467-019-08679-z (PMC6379396; doi:10.1038/s41467-019-08679-z)
Supplement: Supplementary file 1 — Supplementary Information [file 41467_2019_8679_MOESM1_ESM.pdf]

Long non-coding RNAs discriminate the stages and gene regulatory states of the human humoral immune response

Agirre et al.

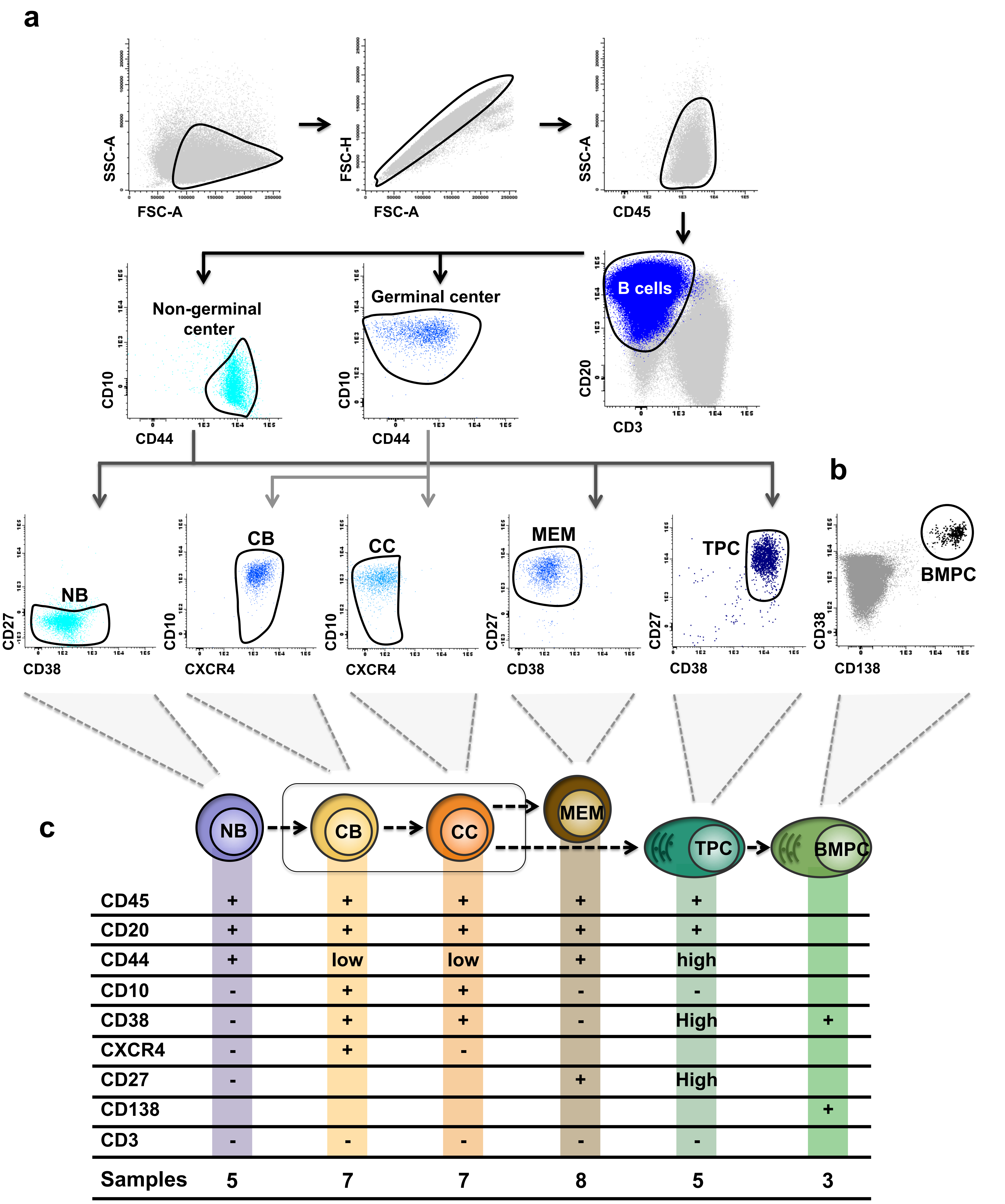

Supplementary Figure 1

Isolation of B cell subpopulations. (a) Gating strategy used for the isolation of naïve B cells (NB), centroblasts (CB), centrocytes (CC), memory B cells (MEM), plasma cells from human tonsils (TPC) and **(b),** plasma cells from bone marrow of healthy donors (BMPC) using multiparameter fluorescence activated cell sorting (FACS). **(c)** Nine different surface antigens were used for the isolation of six B cell subsets by FACS. The number of samples of each type of subsets used for strand specific RNA-seq are indicated at the bottom.

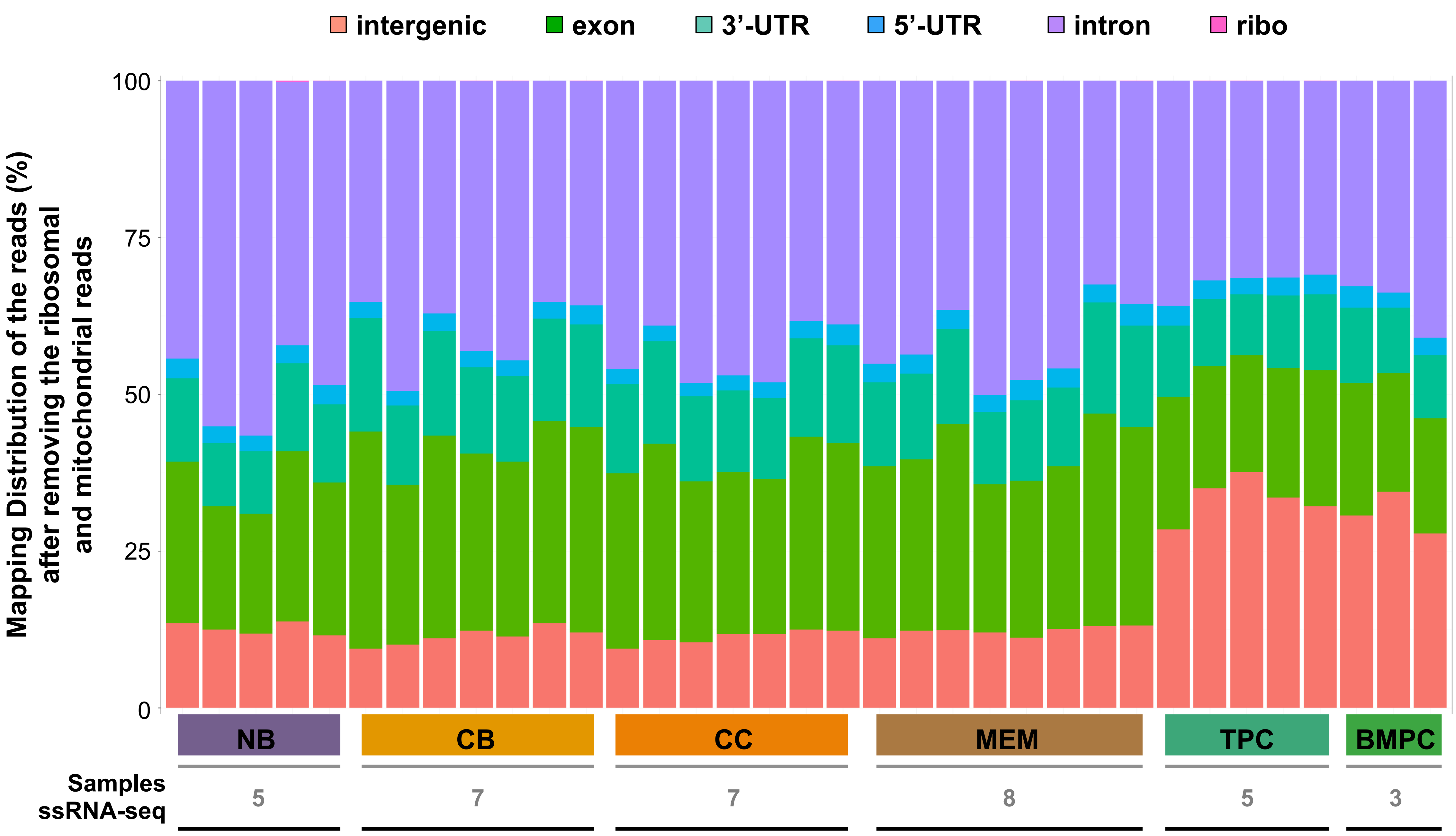

Supplementary Figure 2

Distribution of reads obtained by strand specific RNA-seq strategy in B cell subsets. Mapping distribution of the reads in 5´-UTR, exon, intron, 3´-UTR and intergenic regions of the human genome. NB: Naïve B cells; CB: Centroblasts; CC: Centrocytes; MEM: Memory B cells; TPC: tonsillar plasma cells; BMPC: plasma cells from bone marrow of healthy donors; ssRNA-seq: strand specific RNA sequencing.

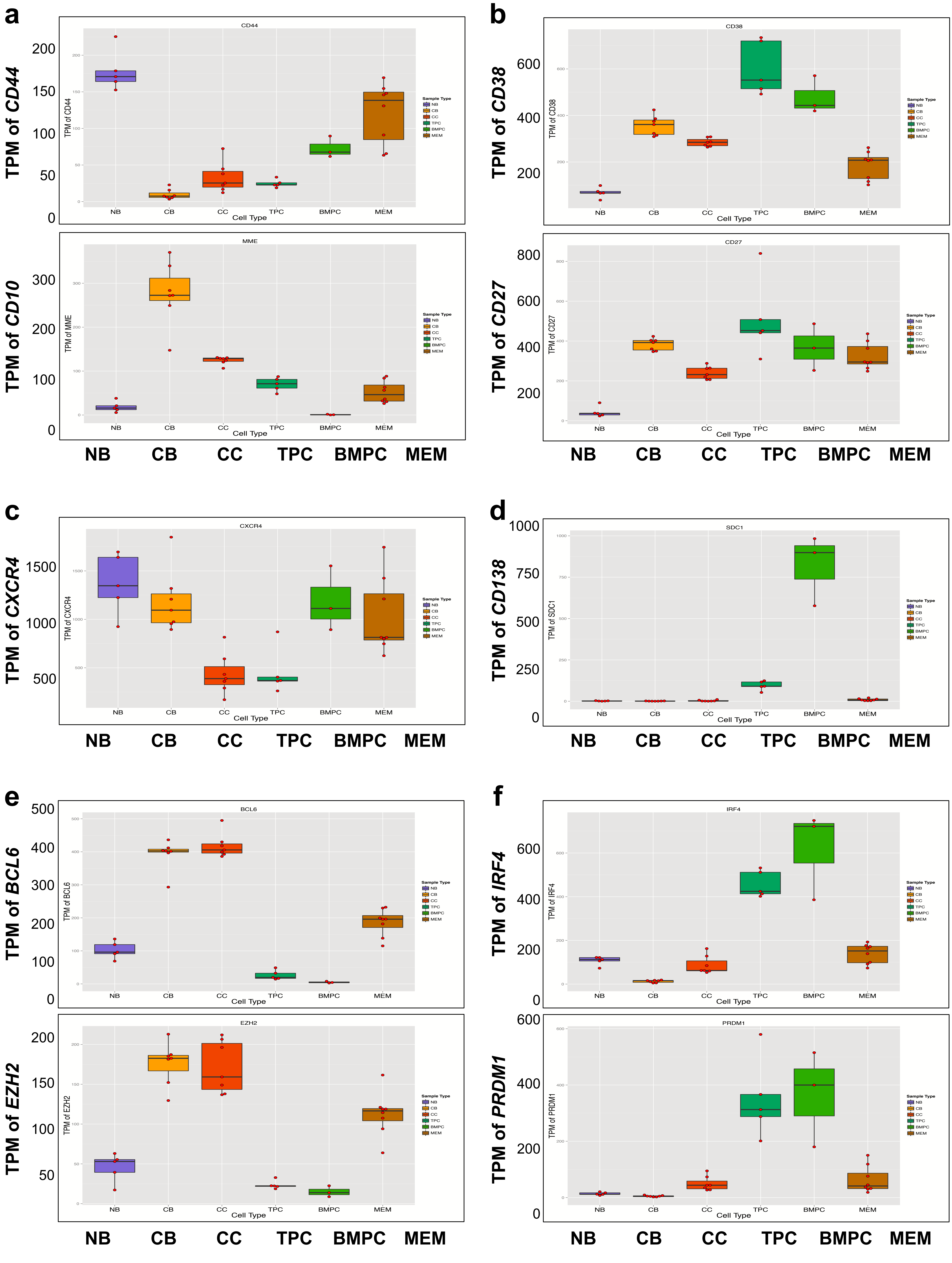

Supplementary Figure 3

Expression of protein specific coding genes of each B cell subsets. (a-d) Box-plot showing the expression of protein coding genes used for the isolation of B cell subpopulations by FACS. **(e)** Box-plot showing the expression of specific protein coding genes of germinal center B cells (*BCL6* and *EZH2*). **(f)** Box-plot showing the expression of specific protein coding genes of plasma cells (*IRF4* and *PRDM1*). The average and deviation between samples are defined. NB: Naïve B cells; CB: Centroblasts; CC: Centrocytes; MEM: Memory B cells; TPC: tonsillar plasma cells; BMPC: plasma cells from bone marrow of healthy donors.

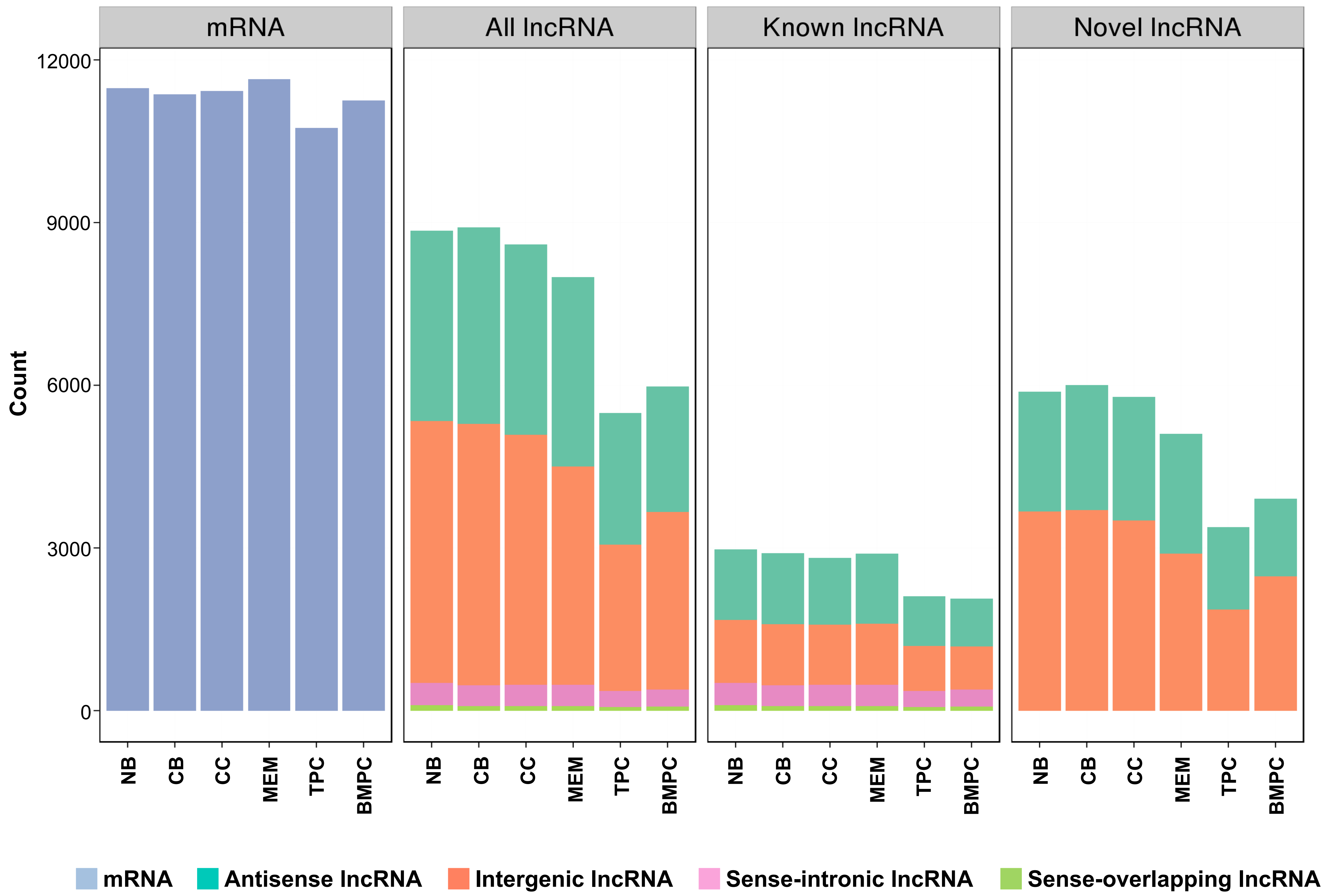

Supplementary Figure 4 Number of lncRNAs and coding genes expressed in each B cell subpopulation. NB: Naïve B cells; CB: Centroblasts; CC: Centrocytes; MEM: Memory B cells; TPC: tonsillar plasma cells; BMPC: plasma cells from bone marrow of healthy donors.

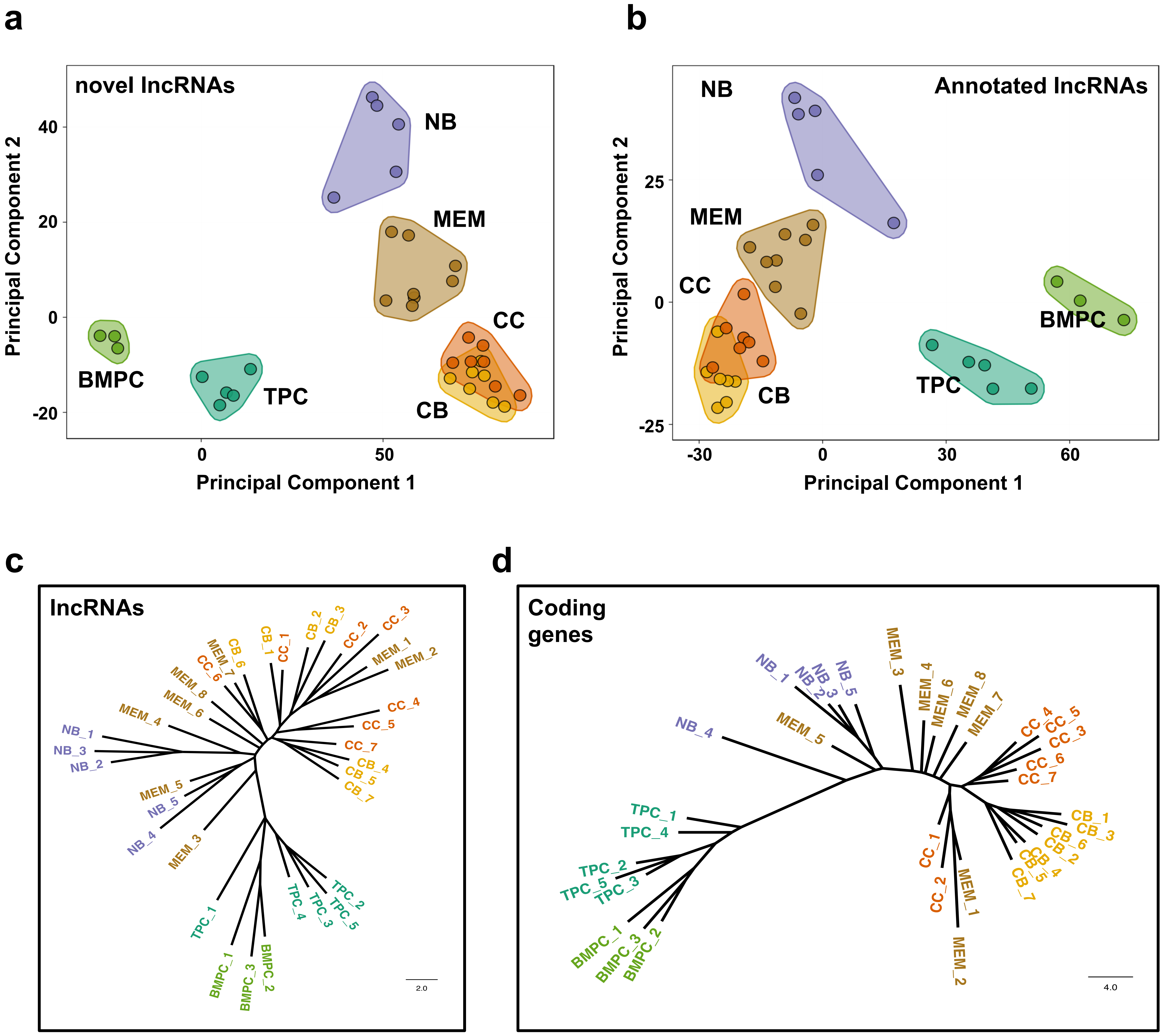

Supplementary Figure 5

lncRNAs of each B cell subsets of human humoral immune response. Unsupervised PCA of RNA-seq data for **(a)** novel lncRNAs and **(b)** previously annotated lncRNAs. Phylogenetic tree for **(c)** lncRNAs and **(d)** protein coding genes of all B cell samples analyzed. NB: Naïve B cells; CB: Centroblasts; CC: Centrocytes; MEM: Memory B cells; TPC: tonsillar plasma cells; BMPC: plasma cells from bone marrow of healthy donors.

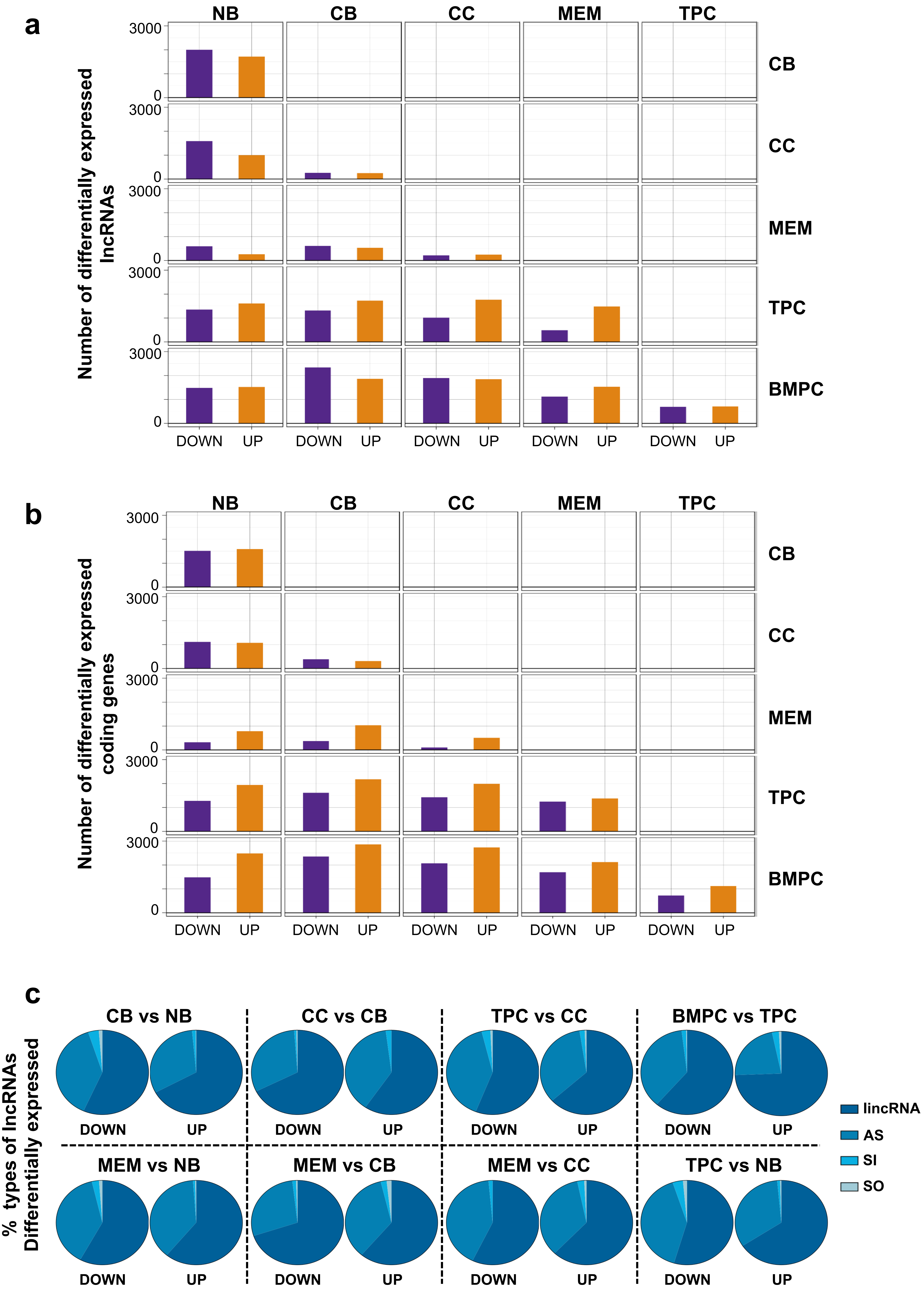

Supplementary Figure 6

Differentially expressed genes during the human humoral immune response. (a) The number of differentially expressed lncRNAs or **(b)** protein coding genes between each B cell type with each of the other B cell subsets. **(c)** Type of lncRNAs differentially expressed. NB: Naïve B cells; CB: Centroblasts; CC: Centrocytes; MEM: Memory B cells; TPC: tonsillar plasma cells; BMPC: plasma cells from bone marrow of healthy donors.

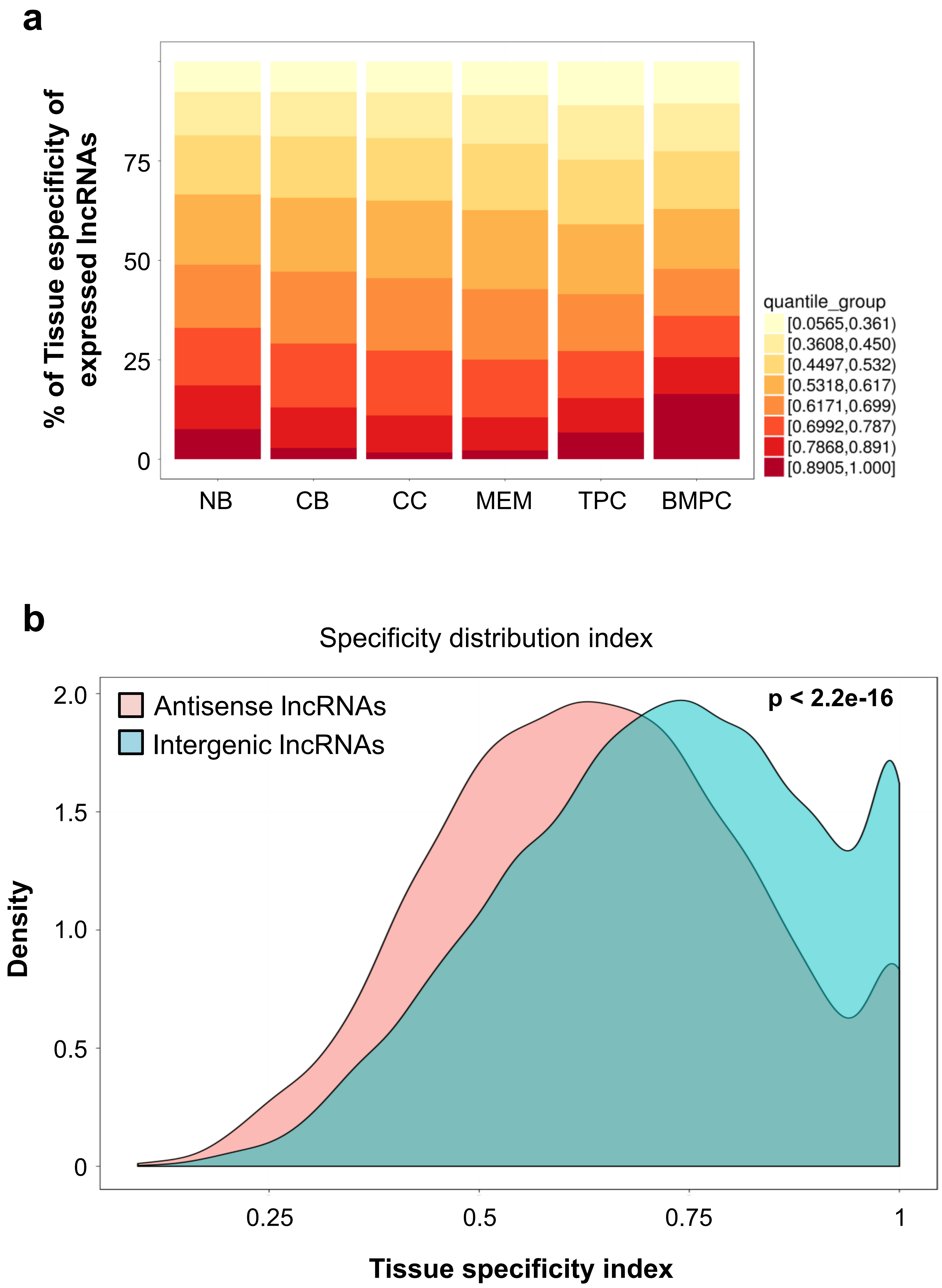

Supplementary Figure 7

Specific expressions of lncRNAs. (a) Tissue specificity index of lncRNAs expressed in each B cell subpopulations. **(b)** Tissue specificity distribution index of intergenic and antisense lncRNAs in B cell subtypes.

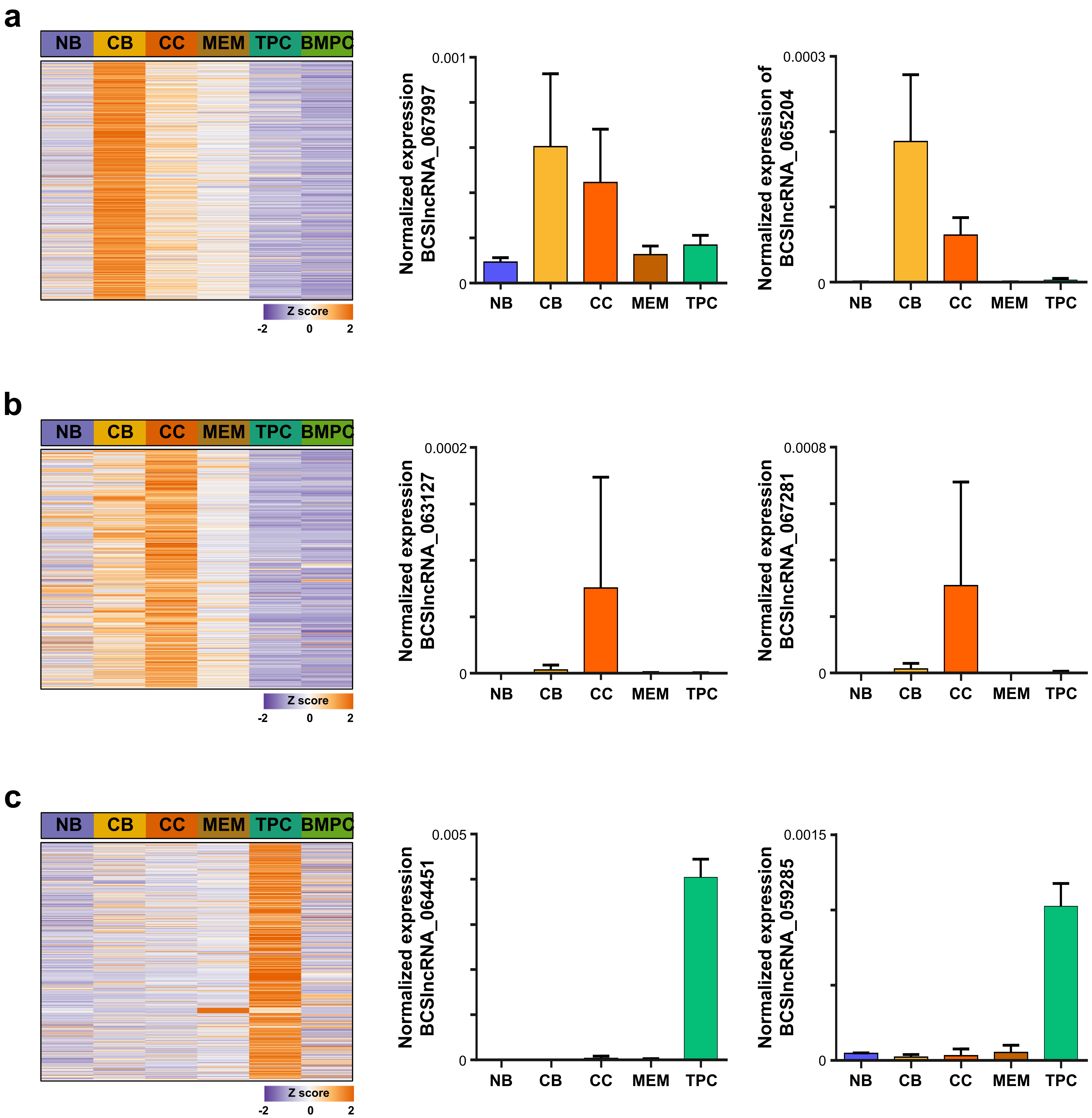

Supplementary Figure 8

Validation of specific lncRNAs of each B cell subsets. (a) Heatmap showing specific expression of lncRNAs in CB cells (left). Q-PCR validation of two of the CB specific lncRNA (right). **(b)** Heatmap showing specific expression of lncRNAs in CC cells (left). Q-PCR validation of two of the CC specific lncRNA (right). **(c)** Heatmap showing specific expression of lncRNAs in TPC (left). Q-PCR validation of two of the plasma cell specific lncRNA (right). The average and deviation between samples are defined. NB: Naïve B cells; CB: Centroblast; CC: Centrocyte; MEM: Memory B cells; TPC: tonsillar plasma cells; BMPC: plasma cells from bone marrow of healthy donors.

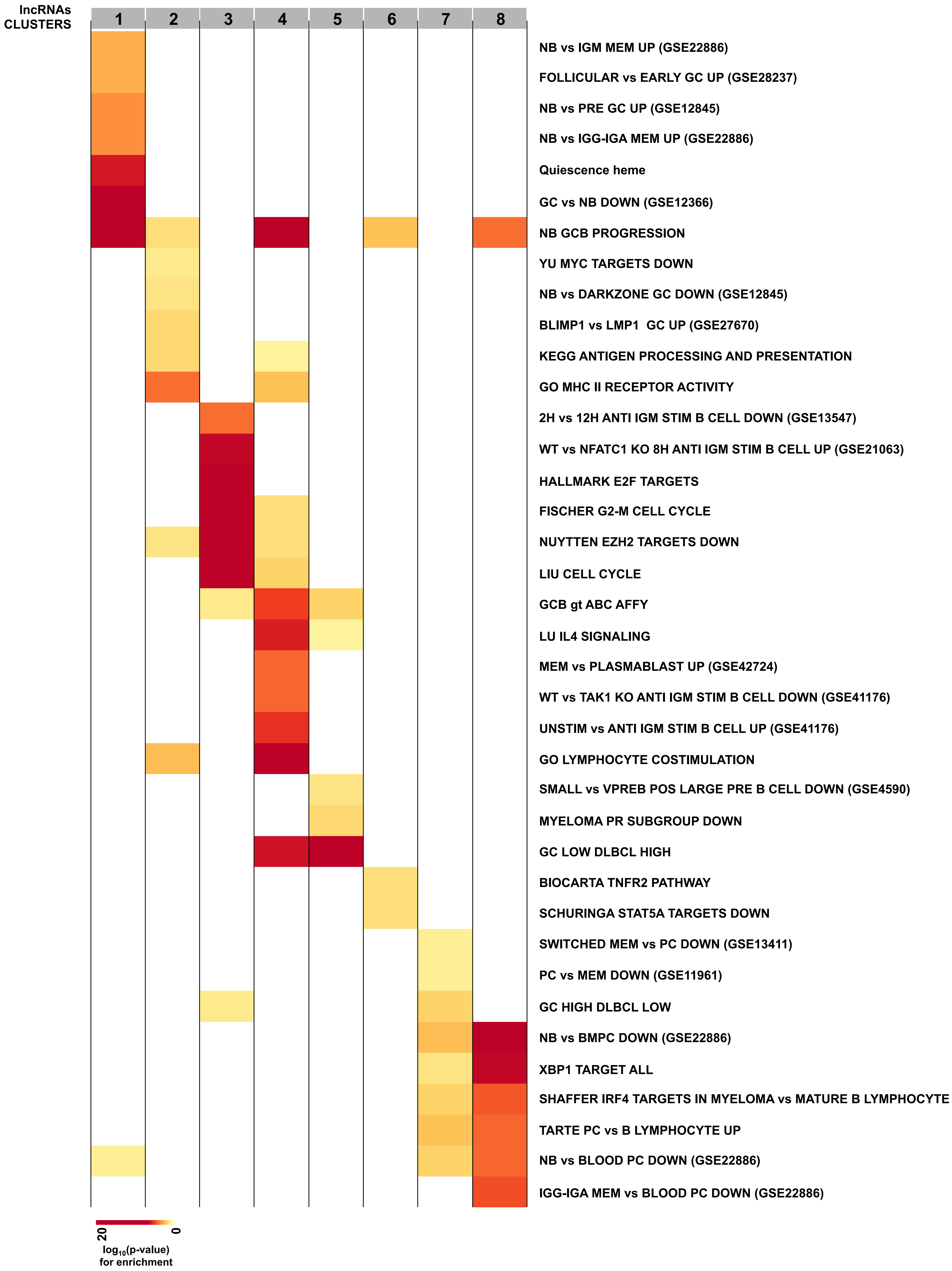

Supplementary Figure 9

Dynamic expressions of lncRNAs during human humoral immune response. (a) Plot showing the protein coding genes that are near to lncRNAs and have known functions in activity and differentiation of the specific cell type.

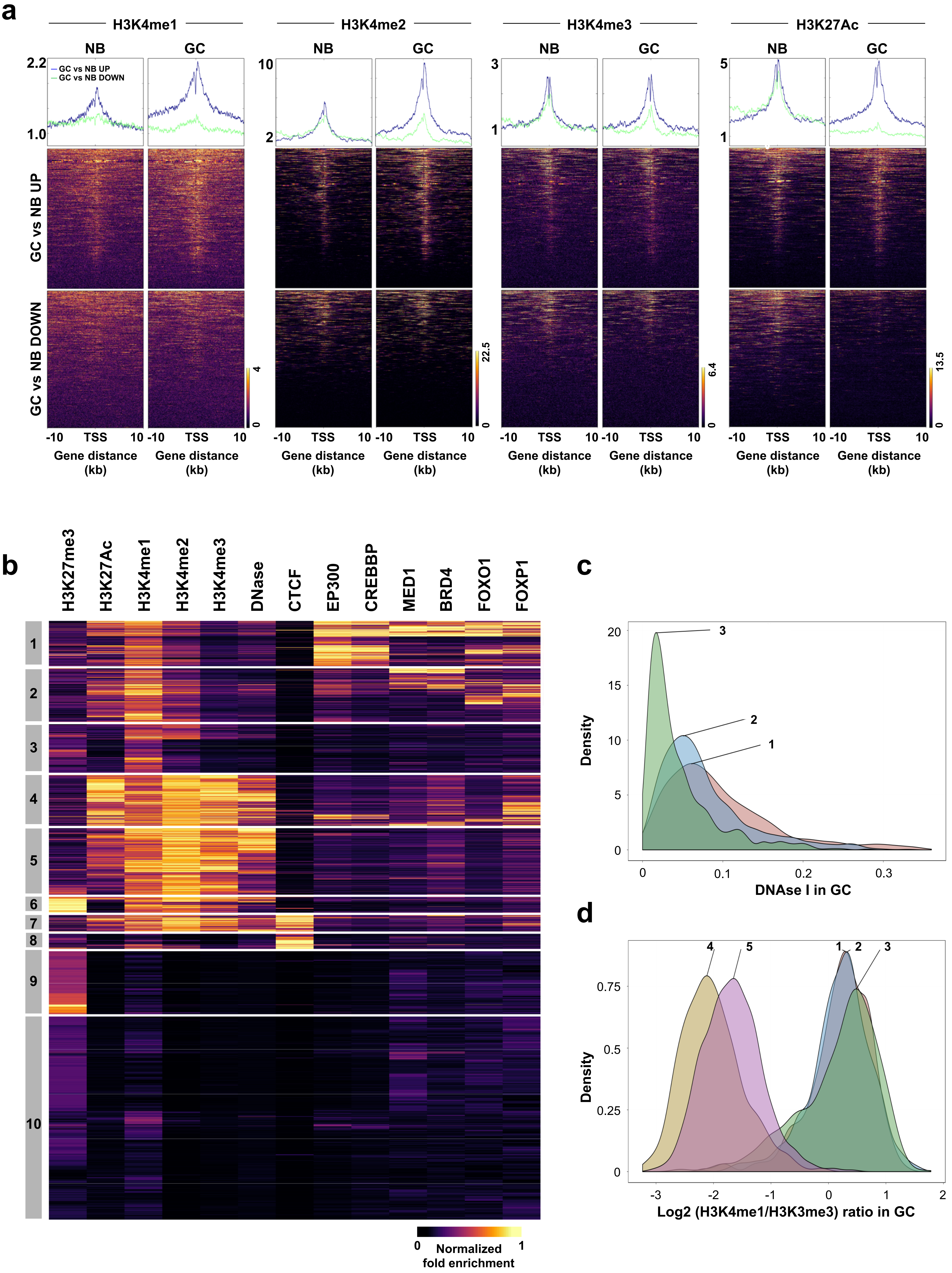

Supplementary Figure 10

Categorization of lncRNAs. (a) Density and tornado plots of ChIP-seq levels of H3K4me1, H3K4me2, H3K4me3 and H3K27ac in lncRNAs differentially expressed between GC and NB cells. **(b)** Heatmap of average of ChIP-seq levels of H3K4me1, H3K4me2, H3K4me3, H3K27ac, H3K27me3, DNase, CTCF, EP300, CREBBP, MED1, BRD4, FOXO1 and FOXP1 in each of the 10 clusters of lncRNAs expressed in GC cells. **(c)** DNAse I enrichment in GC cells into clusters 1, 2 and 3 of lncRNAs. **(d)** H3K4me1/H3K4me3 enrichment ratio in GC cells into cluster 1, 2, 3, 4 and 5 of lncRNAs.

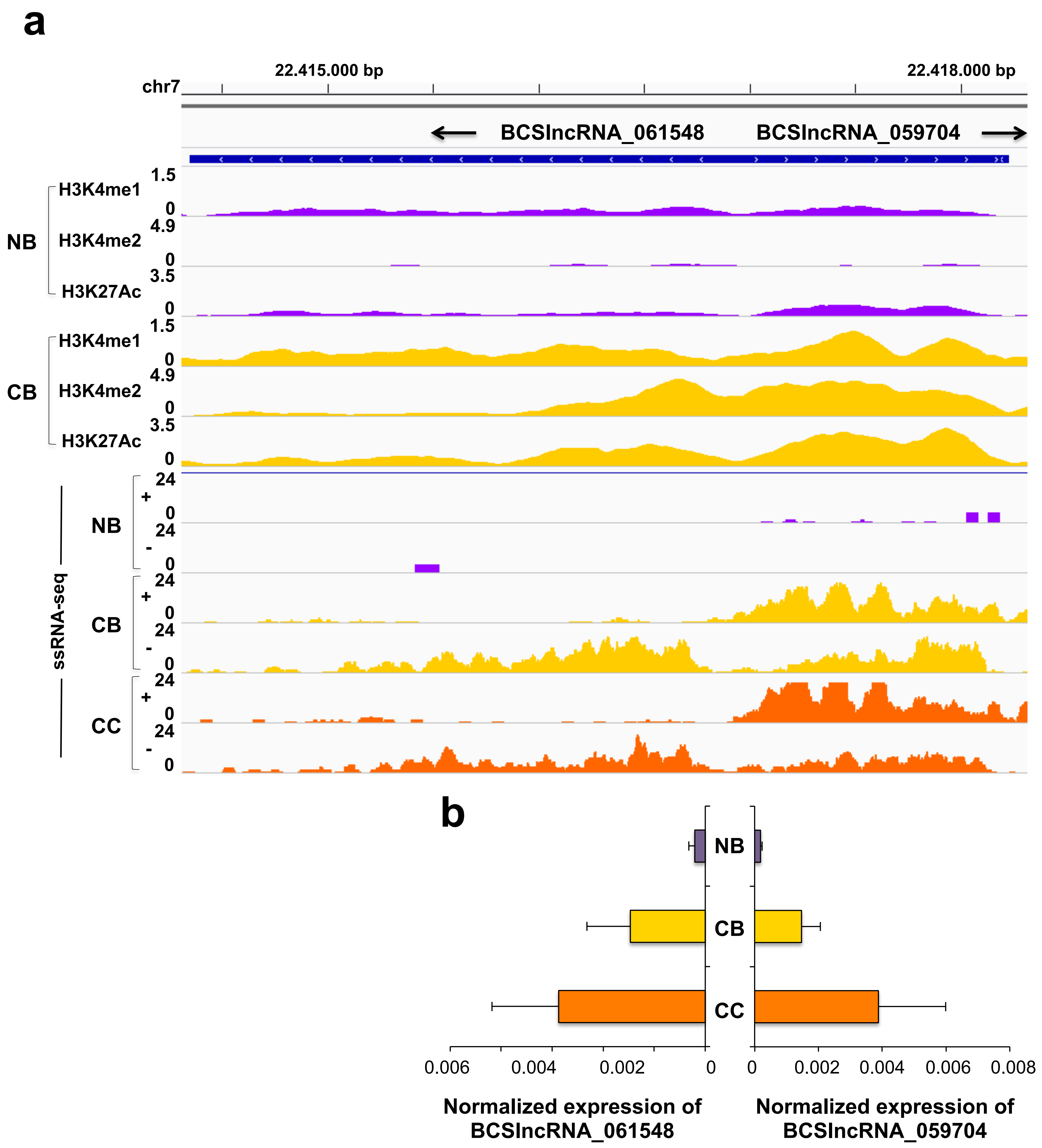

Supplementary Figure 11

Validation of bidirectional enhancer RNAs. a) Genome localization of *BCSlncRNA_061548* and *BCSlncRNA_059704* eRNAs and ChIP-seq levels of H3K4me1, H3K4me2, H3K27Ac and RNA-seq data in this region in CB and NB cells. **b)** Q-PCR validation of *BCSlncRNA_061548* and *BCSlncRNA_059704* eRNAs in NB, CB and CC cells. We used B cells subpopulations isolated from a three new healthy donor tonsils. NB: Naïve B cells; CB: Centroblast and CC: Centrocyte.

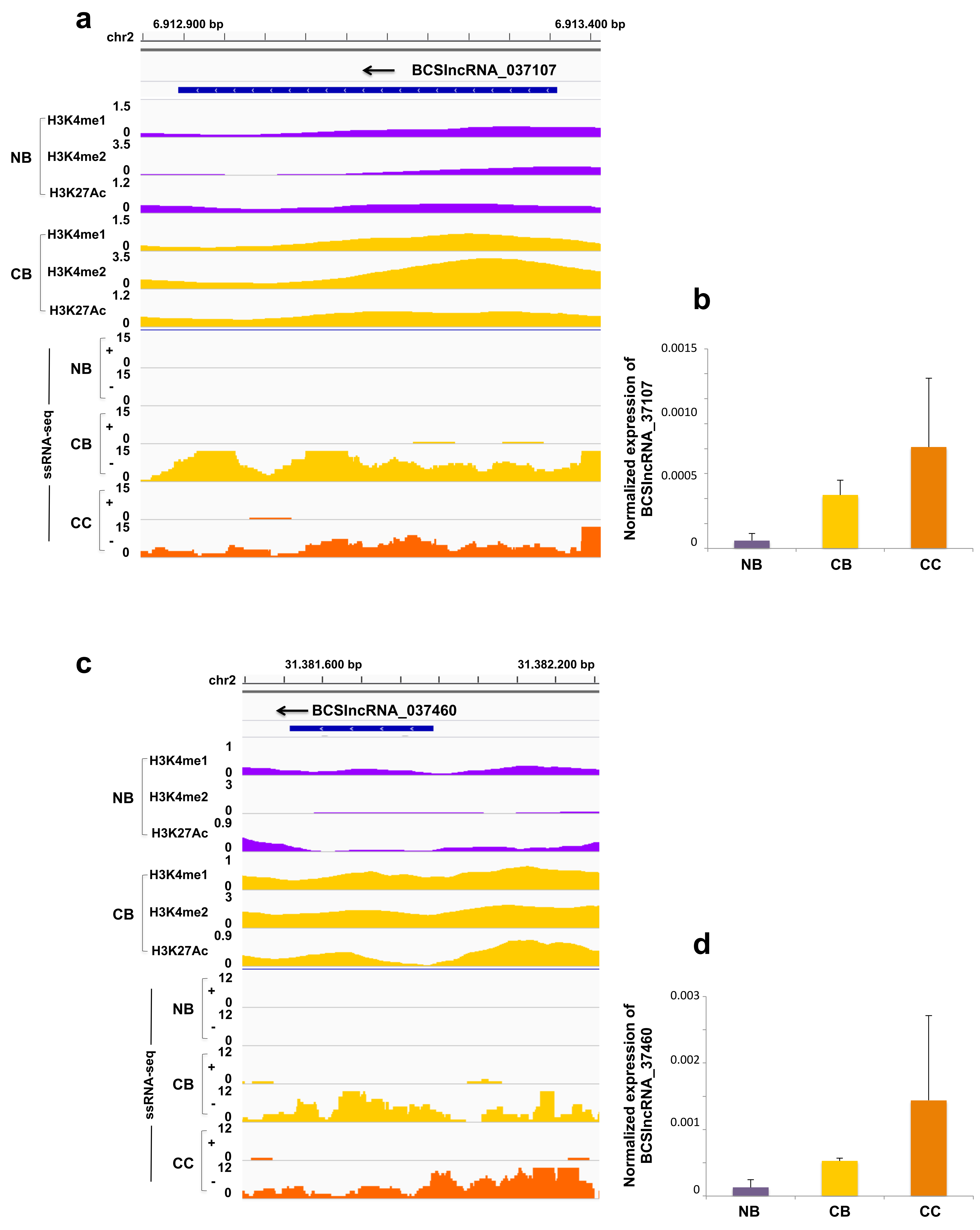

Supplementary Figure 12

Enhancer RNAs expression in NB and CB cells. a-b) Genome localization of *BCSlncRNA_037107* **(a)** and *BCSlncRNA_037460* **(b)** eRNAs and ChIP-seq levels of H3K4me1, H3K4me2, H3K27Ac and RNA-seq data in this region in CB and NB cells. **c-d)** Q-PCR validation of *BCSlncRNA_037107* **(c)** and *BCSlncRNA_037460* **(d)** eRNAs in NB, Cb and CC cells. We used B cells subpopulations isolated from a three new healthy donor tonsils. NB: Naïve B cells; CB: Centroblast and CC: Centrocyte. The average and deviation between samples are defined.

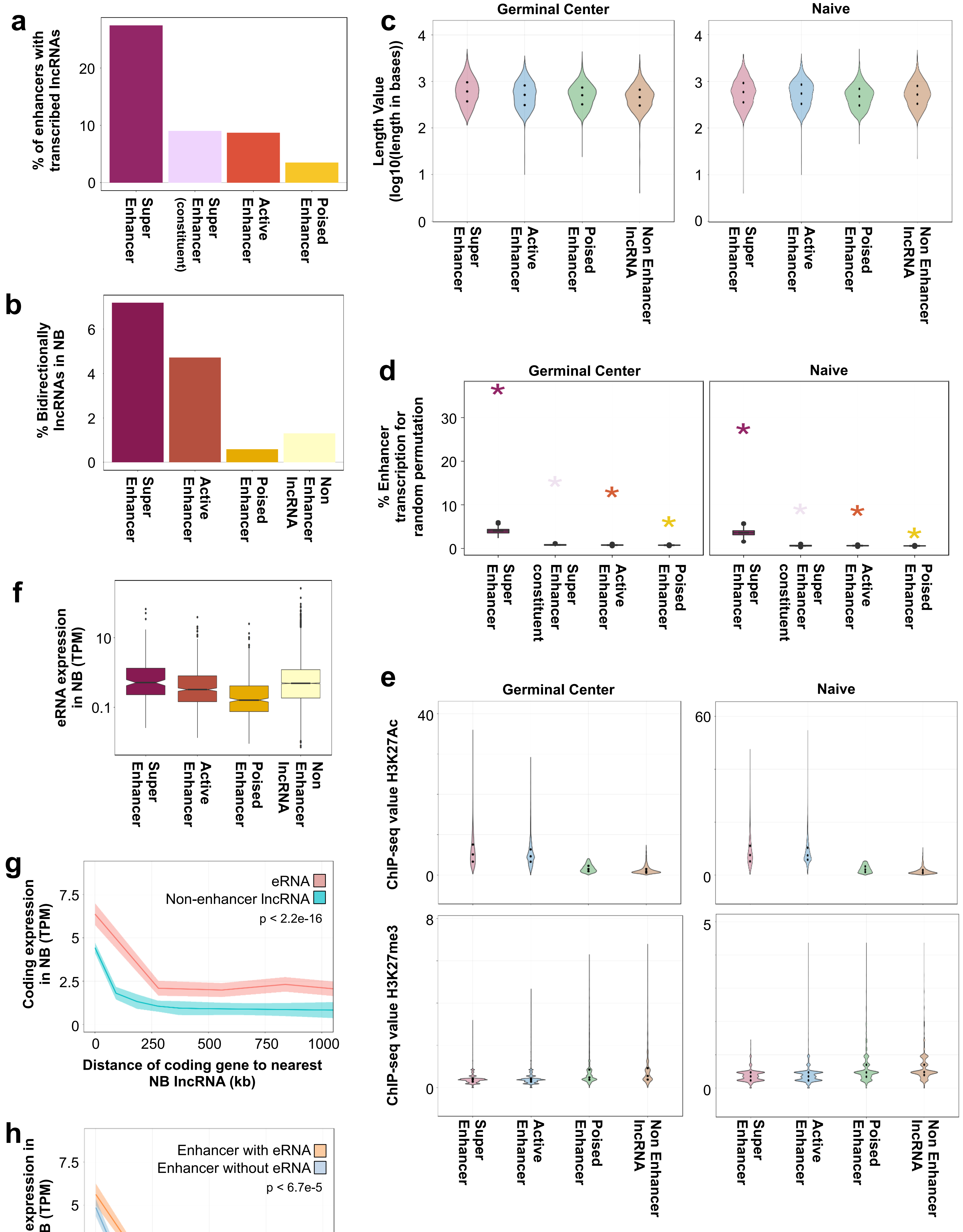

Supplementary Figure 13

Enhancer RNAs in Naïve and Germinal Center B cells. (a) Percentage of enhancer and super enhancer regions with eRNAs transcription in NB. **(b)** Percentage of lncRNAs bi-directionally transcribed from super enhancer regions, active, poised or inactive enhancer regions in NB. **(c)** Length of SE, active, poised and inactive enhancer regions in GC or NB cells. Length value is log10 (length in bases). **(d)** Percentage of transcribed regions for random sampling test. Boxplot shows the fraction of peaks overlapping with a transcribed lncRNAs in 1000 runs of random sampling for the given enhancer types. Asterisks show the real fraction of enhancers that have transcribed lncRNAs. **(e)** H3K27Ac and H3K27me3 levels in super-enhancer, active and poised enhancers detected in GC or NB cells. NB: Naïve B cells; GC: Germinal center. **(f)** eRNAs expression level transcribed from super enhancer regions, active, poised or inactive enhancer regions in NB cells. **(g)** Expression of coding genes nearest to eRNAs or non-enhancer lncRNAs in NB. **(h)** Expression of coding genes nearest to enhancer regions with eRNAs transcription or enhancer regions without eRNAs transcription in NB. NB: Naïve B cells; GC: Germinal center. The average and deviation between samples are defined.

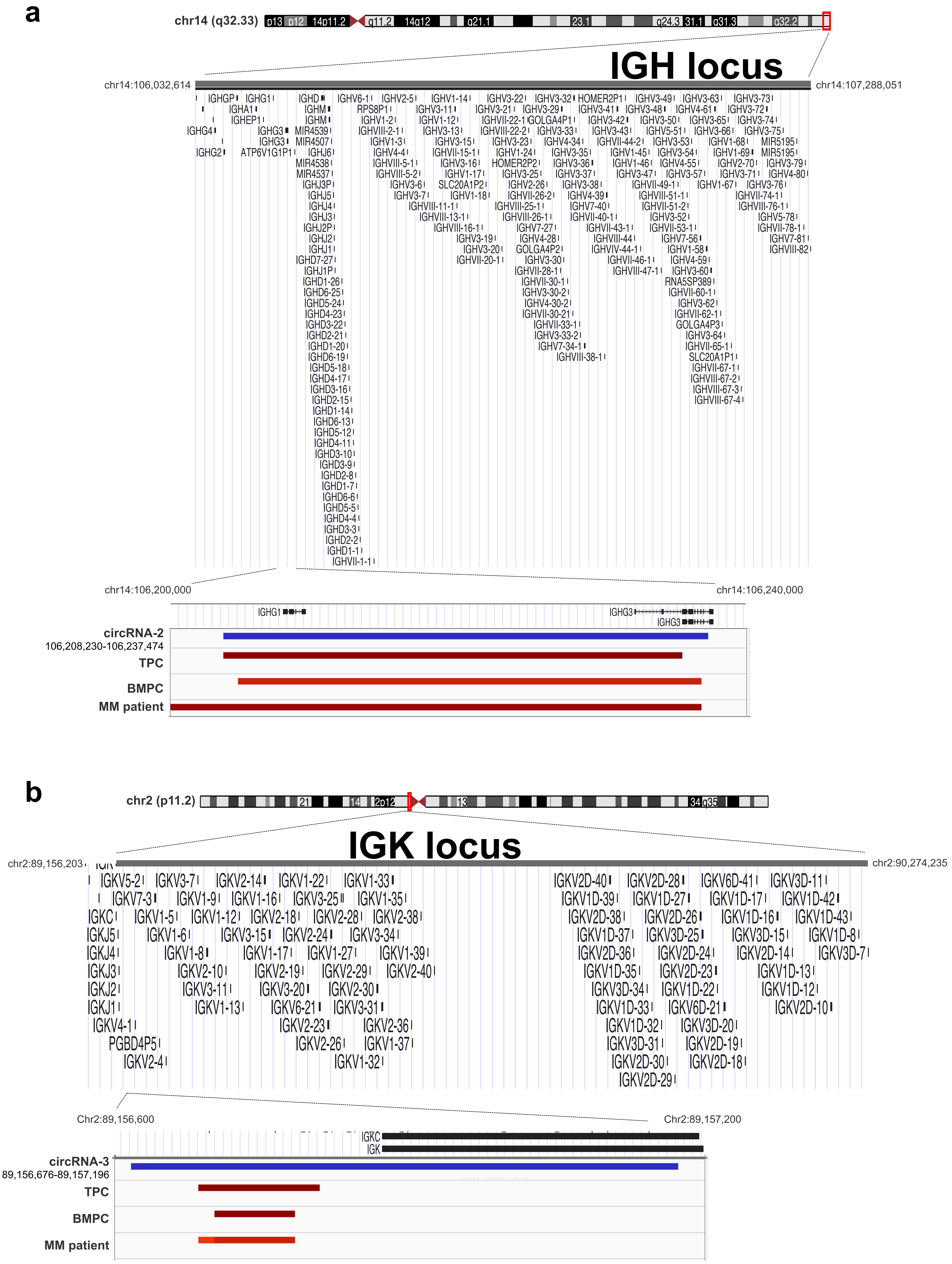

Supplementary Figure 14

Representation of junction reads of *circRNA-2* and *circRNA-3* plasma cells. (a) IGH locus representation and junction reads detected in this locus for *circRNA-2* in plasma cells. **(b)** IGK locus representation and junction reads detected in this locus for *circRNA-3* in plasma cells. TPC: tonsilar plasma cells; BMPC: plasma cells obtained from bone marrow and MM: multiple myeloma.

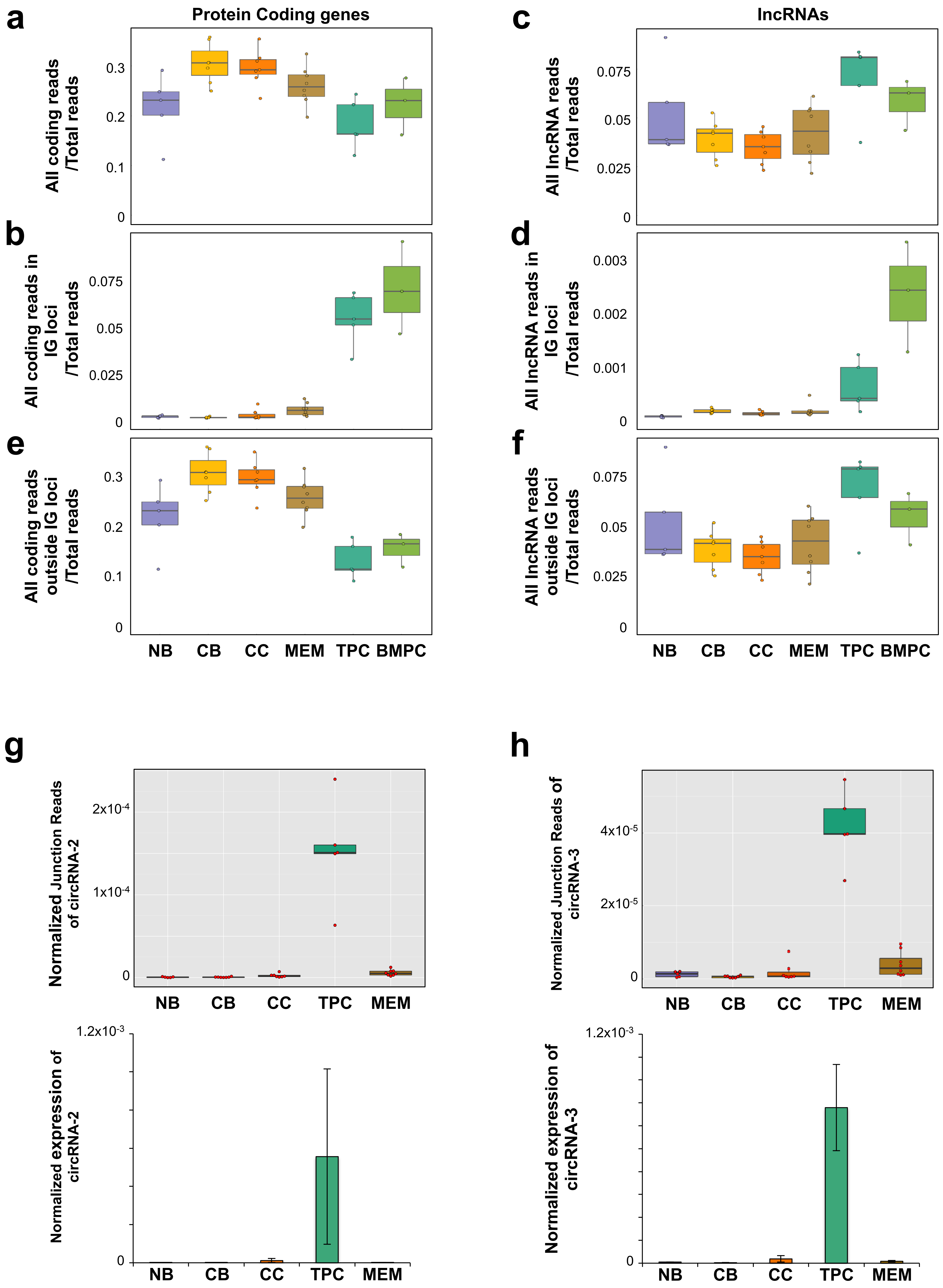

Supplementary Figure 15

circRNAs in different subpopulations of B cell. (a) Box plots showing the average expression of total junction reads from coding genes. **(b)** Box plots showing the average expression of junction reads of coding genes from Ig genes. **(c)** Box plots showing the average expression of total junction reads from lncRNAs. **(d)** Box plots showing the average expression of total junction reads of lncRNAs from Ig genes. **(e)** Box plots showing the average expression of junction reads of coding genes from non-Ig genes. **(f)** Box plots showing the average expression of junction reads of lncRNAs from non-Ig genes. **(g, h)** TPM values and Q-PCR validation of circRNAs derived from Ig genes. NB: Naïve B cells; CB: Centroblasts; CC: Centrocytes; MEM: Memory B cells; TPC: tonsillar plasma cells; BMPC: plasma cells from bone marrow of healthy donors; Ig: Immunoglobulin genes. The average and deviation between samples are defined.

Supplementary Table 1: **Q-RT-PCR primers used for the validation of specific novel and annotated lncRNAs, eRNAs and circRNAs.**

	LEFT PRIMER	RIGHT PRIMER
**BCSlncRNA_067997**	GAAGCGAGGAAAGAGATAGGG	GCTTGCAGTGCGTTTCATTA
**BCSlncRNA_063127**	GGACTCCAGAGCACGTTAGC	AAGCAACACTGGGAATCACC
**RP11-80H8.3**	GCACACTGGACAGAGCTGACT	CCCCAAAGTGAGTAGGGATG
**BCSlncRNA_061548**	TCAAGTGCCTGGGCTATTTT	GGACAACAAGAAGGGAAGCA
**BCSlncRNA_059704**	TTTTTCCCGAAGTCAGCTTG	GCCCATCAAGATTTCAGCTC
**circRNA-2**	CTGGCCTCTCACCAACTCTC	TGCAAGGTCTCCAACAAAGC
**circRNA-3**	GCGGGAAGATGAAGACAGAT	TGCTAATGTTGGAGGAGAATGA
