# Supplementary material for: Long non-coding RNAs discriminate the stages and gene regulatory states of human humoral immune response
Source: Nat Commun. 2019 Feb 18;10:821. doi: 10.1038/s41467-019-08679-z (PMC6379396; doi:10.1038/s41467-019-08679-z)
Supplement: Supplementary file 3 — Description of Additional Supplementary Files [file 41467_2019_8679_MOESM3_ESM.pdf]

Description of Additional Supplementary Files

Supplementary Data 1
GTF file of lncRNA annotation

Supplementary Data 2
Overview of lncRNA coordinates and clustering for expression patterns through B-cell differentiation.

Supplementary Data3
Differentially expressed coding genes and lncRNAs between each stage.

Supplementary Data 4
Enriched pathways from DEG clusters.

Supplementary Data 5
Chromatin mark clusters, enhancer status and eRNAs in NB & GC.

Supplementary Data 6
Coding genes close to superenhancers in NB and GC cells.

Supplementary Data 7
Significant biological pathways for coding genes near eRNAs.

Supplementary Data 8
circRNAs in humoral immune response.
