# Supplementary material for: Long non-coding RNAs discriminate the stages and gene regulatory states of human humoral immune response
Source: Nat Commun. 2019 Feb 18;10:821. doi: 10.1038/s41467-019-08679-z (PMC6379396; doi:10.1038/s41467-019-08679-z)
Supplement: Supplementary file 12 — Reporting Summary [file 41467_2019_8679_MOESM12_ESM.pdf]

nature research

Corresponding author(s): *Double-blind peer review submissions: write DBPR and your manuscript number here instead of author names.*

Last updated by author(s): *YYYY-MM-DD*

Reporting Summary

Nature Research wishes to improve the reproducibility of the work that we publish. This form provides structure for consistency and transparency in reporting. For further information on Nature Research policies, see Authors & Referees and the Editorial Policy Checklist.

Statistics

For all statistical analyses, confirm that the following items are present in the figure legend, table legend, main text, or Methods section.

n/a	Confirmed	
☐	☒	The exact sample size (n) for each experimental group/condition, given as a discrete number and unit of measurement
☐	☒	A statement on whether measurements were taken from distinct samples or whether the same sample was measured repeatedly
☐	☒	The statistical test(s) used AND whether they are one- or two-sided<br>*Only common tests should be described solely by name; describe more complex techniques in the Methods section.*
☐	☒	A description of all covariates tested
☐	☒	A description of any assumptions or corrections, such as tests of normality and adjustment for multiple comparisons
☐	☒	A full description of the statistical parameters including central tendency (e.g. means) or other basic estimates (e.g. regression coefficient) AND variation (e.g. standard deviation) or associated estimates of uncertainty (e.g. confidence intervals)
☒	☐	For null hypothesis testing, the test statistic (e.g. F, t, r) with confidence intervals, effect sizes, degrees of freedom and P value noted<br>*Give P values as exact values whenever suitable.*
☒	☐	For Bayesian analysis, information on the choice of priors and Markov chain Monte Carlo settings
☒	☐	For hierarchical and complex designs, identification of the appropriate level for tests and full reporting of outcomes
☒	☐	Estimates of effect sizes (e.g. Cohen's d, Pearson's r), indicating how they were calculated

Our web collection on statistics for biologists contains articles on many of the points above.

Software and code

Policy information about availability of computer code

Data collection	*Provide a description of all commercial, open source and custom code used to collect the data in this study, specifying the version used OR state that no software was used.*
Data analysis	R, STAR, Cufflinks, Subread featureCounts, PhyloCSF, BWA, MACS, CIRI

For manuscripts utilizing custom algorithms or software that are central to the research but not yet described in published literature, software must be made available to editors/reviewers. We strongly encourage code deposition in a community repository (e.g. GitHub). See the Nature Research guidelines for submitting code & software for further information.

Data

Policy information about availability of data

All manuscripts must include a data availability statement. This statement should provide the following information, where applicable:

- Accession codes, unique identifiers, or web links for publicly available datasets
- A list of figures that have associated raw data
- A description of any restrictions on data availability

Data generated for this study has been made available in NCBI GEO with accession GSE114816 (reviewer access token: ctilcsoynbwzjup).
Other data used for this study can be found under accessions GSE114803, GSE45982, GSE84022, GSE68349, GSE53601 and ENCSR000EIZ.

Field-specific reporting

Please select the one below that is the best fit for your research. If you are not sure, read the appropriate sections before making your selection.

☒ Life sciences ☐ Behavioural & social sciences ☐ Ecological, evolutionary & environmental sciences

For a reference copy of the document with all sections, see nature.com/documents/nr-reporting-summary-flat.pdf

Life sciences study design

All studies must disclose on these points even when the disclosure is negative.

Sample size	no sample size calculation was performed
Data exclusions	2 subjects were excluded from the analysis after comparing the inter-group distance of samples to the group centroid and applying Chauvenet's criterion. 1 sample that failed sequencing QC due to very low coverage was removed.
Replication	All attempts at replication were successful
Randomization	This is not relevant in our study
Blinding	Blinding was not relevant

Reporting for specific materials, systems and methods

We require information from authors about some types of materials, experimental systems and methods used in many studies. Here, indicate whether each material, system or method listed is relevant to your study. If you are not sure if a list item applies to your research, read the appropriate section before selecting a response.

Materials & experimental systems

n/a	Involved in the study	
☐	☒	Antibodies
☒	☐	Eukaryotic cell lines
☒	☐	Palaeontology
☒	☐	Animals and other organisms
☐	☒	Human research participants
☒	☐	Clinical data

Methods

n/a	Involved in the study	
☐	☒	ChIP-seq
☐	☒	Flow cytometry
☒	☐	MRI-based neuroimaging

Antibodies

Antibodies used	The following monoclonal antibody (MoAb) combination was used for the cell isolation from tonsils: CD45-OC515 (Clone HI30, Immunostep, Salamanca, Spain); CD20-Pacific Blue (Clone 2H7, Bioloegend, San Diego, California, UnitedStates); CD44-APCH7 (Clone G44-26, Beckton Dickinson, Dur-ham, North Carolina, United States). CD10 PE-Cy7 (Clone HI10a BecktonDickinson, Durham, North Carolina, United States); CD38-FITC (Clone LD38, Cytognos, Salamanca, Spain); CXCR4-PE (Clone 12G5, Beckton Dickinson, Durham, North Carolina, UnitedStates); CD27-APC (Clone L128, Beckton Dickinson, Durham, North Carolina, UnitedStates) and CD3-PerCP-Cy5.5 (Clone SK7, Beckton Dickinson, Durham, North Carolina, United States). BMPC were fluorescence-activated cell sorted (FACS) (FACSAria II, Becton Dickinson Biosciences, Durham, North Carolina, United States) from human bone marrow of healthy donors using CD38-FITC (Clone LD38, Cytognos, Salamanca, Spain); CD138-BV421 (Clone MI15, Beckton Dickinson, Dur-ham, North Carolina, United States) and CD27-BV510 (Clone 0323, Bioloegend, San Diego, California, UnitedStates).
Validation	These antibodies have been validated by the EuroFlow consortium for the diagnosis and monitoring of leukemia and lymphoma. The flow cytometry laboratory is member of EuroFlow.

Human research participants

Policy information about studies involving human research participants

Population characteristics	We used the tonsils and bone marrow from healthy donors. Age: 20-25 years.
Recruitment	All participants were recruited at the University Clinic of Navarra following all the ethical aspects of the Biobank of the University of Navarra.

Ethics oversight	Clínica Universidad de Navarra

Note that full information on the approval of the study protocol must also be provided in the manuscript.

ChIP-seq

Data deposition

☒ Confirm that both raw and final processed data have been deposited in a public database such as GEO.

☒ Confirm that you have deposited or provided access to graph files (e.g. BED files) for the called peaks.

Data access links<br>*May remain private before publication.*	ChIP-seq data is from "Jiang Y, CREBBP inactivation promotes the development of HDAC3-dependent lymphomas, Cancer Discovery 2017". Data is available in GSE114803 (reviewer access token: sfkhamsavzahzcp), GSE45982, GSE84022, GSE68349 and GSE53601.
Files in database submission	As listed in GEO (RNAseq and ChIPseq)
Genome browser session<br>(e.g. UCSC)	*Provide a link to an anonymized genome browser session for "Initial submission" and "Revised version" documents only, to enable peer review. Write "no longer applicable" for "Final submission" documents.*

Methodology

Replicates	All methodology as defined in:<br>Jiang Y, CREBBP inactivation promotes the development of HDAC3-dependent lymphomas, Cancer Discovery 2017
Sequencing depth	*Describe the sequencing depth for each experiment, providing the total number of reads, uniquely mapped reads, length of reads and whether they were paired- or single-end.*
Antibodies	*Describe the antibodies used for the ChIP-seq experiments; as applicable, provide supplier name, catalog number, clone name, and lot number.*
Peak calling parameters	*Specify the command line program and parameters used for read mapping and peak calling, including the ChIP, control and index files used.*
Data quality	*Describe the methods used to ensure data quality in full detail, including how many peaks are at FDR 5% and above 5-fold enrichment.*
Software	*Describe the software used to collect and analyze the ChIP-seq data. For custom code that has been deposited into a community repository, provide accession details.*

Flow Cytometry

Plots

Confirm that:

☒ The axis labels state the marker and fluorochrome used (e.g. CD4-FITC).

☒ The axis scales are clearly visible. Include numbers along axes only for bottom left plot of group (a 'group' is an analysis of identical markers).

☒ All plots are contour plots with outliers or pseudocolor plots.

☒ A numerical value for number of cells or percentage (with statistics) is provided.

Methodology

Sample preparation	Naive B cells (NB), Centroblast (CB), Centrocyte (CC), Memory (MEM) and plasma cells (TPC) were isolated from human tonsils of healthy donors and bone marrow plasma cells (BMPC) from bone marrow of healthy donors by multiparameter fluorescence-activated cell sorting (FACS) using the expression level of 9 different surface antigens as previously describe (Pascual M. 2017).
Instrument	FACSAria II, Becton Dickinson Biosciences, San Jose, California, United States
Software	FACSDiva 6.1, Becton Dickinson Biosciences, San Jose, California, United States
Cell population abundance	The abundance varies according to cell type and tissue (e.g.: plasma cells are more abundant in tonsils as compared to bone marrow). Purity was 97% or greater in all sorted cell types.
Gating strategy	*Describe the gating strategy used for all relevant experiments, specifying the preliminary FSC/SSC gates of the starting cell population, indicating where boundaries between "positive" and "negative" staining cell populations are defined.*

☒ Tick this box to confirm that a figure exemplifying the gating strategy is provided in the Supplementary Information.
