# Supplementary material for: Long non-coding RNAs discriminate the stages and gene regulatory states of human humoral immune response
Source: Nat Commun. 2019 Feb 18;10:821. doi: 10.1038/s41467-019-08679-z (PMC6379396; doi:10.1038/s41467-019-08679-z)
Supplement: Supplementary file 2 — Peer Review File [file 41467_2019_8679_MOESM2_ESM.pdf]

Editorial Note: This manuscript has been previously reviewed at another journal that is not operating a transparent peer review scheme. This document only contains reviewer comments and rebuttal letters for versions considered at Nature Communications.

REVIEWERS' COMMENTS:

Reviewer #1 (Remarks to the Author):

The authors adequately revised this manuscript and satisfactorily addressed my earlier comments.

Reviewer #2 (Remarks to the Author):

The authors have addressed my comments to satisfaction. I think this is a thorough and well-performed study, which will be a great resource to the immunology community.

I have no additional comments.
